# A review of cardiac troponin I detection by surface enhanced Raman spectroscopy: Under the spotlight of point-of-care testing

**DOI:** 10.3389/fchem.2022.1017305

**Published:** 2022-10-13

**Authors:** Anel I. Saviñon-Flores, Fernanda Saviñon-Flores, G. Trejo, Erika Méndez, Ştefan Ţălu, Miguel A. González-Fuentes, Alia Méndez-Albores

**Affiliations:** ^1^ Centro de Química-ICUAP- Posgrado en Ciencias Ambientales, Benemérita Universidad Autónoma de Puebla, Puebla, Mexico; ^2^ Facultad de Ciencias Químicas, Benemérita Universidad Autónoma de Puebla, Puebla, Mexico; ^3^ Laboratory of Composite Materials and Functional Coatings, Center for Research and Technological Development in Electrochemistry (CIDETEQ), Querétaro, Mexico; ^4^ Technical University of Cluj-Napoca, The Directorate of Research, Development and Innovation Management (DMCDI), Cluj-Napoca, Romania

**Keywords:** cardiac troponin I (cTnI), acute myocardial infarction (AMI), point-of-care testing, surface-enhanced Raman spectroscopy (SERS), SERS-based immunoassays

## Abstract

Cardiac troponin I (cTnI) is a biomarker widely related to acute myocardial infarction (AMI), one of the leading causes of death around the world. Point-of-care testing (POCT) of cTnI not only demands a short turnaround time for its detection but the highest accuracy levels to set expeditious and adequate clinical decisions. The analytical technique Surface-enhanced Raman spectroscopy (SERS) possesses several properties that tailor to the POCT format, such as its flexibility to couple with rapid assay platforms like microfluidics and paper-based immunoassays. Here, we analyze the strategies used for the detection of cTnI by SERS considering POCT requirements. From the detection ranges reported in the reviewed literature, we suggest the diseases other than AMI that could be diagnosed with this technique. For this, a section with information about cardiac and non-cardiac diseases with cTnI release, including their release kinetics or cut-off values are presented. Likewise, POCT features, the use of SERS as a POCT technique, and the biochemistry of cTnI are discussed. The information provided in this review allowed the identification of strengths and lacks of the available SERS-based point-of-care tests for cTnI and the disclosing of requirements for future assays design.

## 1 Introduction

Since 2000 and continuously until today, cardiovascular diseases (CVD) are the main cause of mortality and morbidity in the world (World Health Organization, 2000). The above, despite the great advances in the treatment and management of these diseases, such as the introduction of first totally implantable pacemaker in 1958 (Van Hemel and Van der Wall, 2008), the first heart transplant performed by Dr. Christian Barnard in 1967 (Brink and Hassoulas, 2009), the coronary angioplasty to treat patients with stenosis in 1974 (Grüntzig and Hopff, 1974), the development of bioabsorbable stents coated with antiproliferative drugs to treat arteriosclerosis in 1999 (Tamai et al., 1999), the remote monitoring for the effective management of heart failure in 2008 (Santini et al., 2008), and the development of gene therapies as treatment for CVD since 2017 (Ylä-Herttuala et al., 2017).

Today, CVD represent a serious public health problem (World Heart Federation, 2022) and have been included in goal 3.4 of the Sustainable Development Goals (SDG) of the United Nations 2030 Agenda in 2015, where it was established to reduce premature mortality from non-communicable diseases by 33%, including CVD (World Health Organization, 2016). Since then, several international organizations such as the World Health Organization (WHO), the Pan American Health Organization (PAHO), the World Heart Federation (WHF) and the United Nations (UN) have joined this effort. The above, through the implementation of various strategies that include: 1) the promotion of healthy environments and lifestyles impact on the prevention and control of these diseases, 2) the accessibility to the diagnosis and treatment of CVD from the primary health-care levels through the updating and improvement of health services, and 3) the development of new techniques, technologies, preventive diagnostic, and therapeutic procedures, promoting health research and innovation (World Health Organization, 2016; Pan American Health Organization, 2020; United Nations Sustainable Development, 2022; World Heart Federation, 2022).

CVD are a heterogeneous group of diseases that affect both the circulatory system and the heart muscle. AMI, cerebrovascular accidents (CVA), ischemic heart disease (IC), heart failure (HF), and unstable angina pectoris (UAP) are considered the most frequent CVD (World Health Organization, 2022; World Heart Federation, 2022). These pathologies arise due to various causes, which in turn originate from complex interactions between their risk factors. Currently, CVD risk factors fall into four main categories: 1) biological factors, including modifiable (obesity, diabetes, hypertension, cancer, and chronic respiratory diseases) and non-modifiable (age, gender, ethnicity, and genetic predisposition), 2) behavioral factors (eating habits, sedentary lifestyle, stress, smoking/alcoholism), 3) environmental factors (anthropogenic pollution, unhealthy conditions, socio-economic development, epidemics and pandemics), and 4) global influencing factors (urbanism, technological development, migration, international policies) (Cosselman et al., 2015; Bhatnagar, 2017). In this way, the treatment, control, and prevention of CVD must be tackled with a multidisciplinary focus, because the human environments where everyone develops are complex and the key determinants are highly variable.

A good diagnosis has an impact on adequate intervention, on timely treatment and on the proper management and control of CVD. In turn, it increases the prognosis of life and reduces complications in patients. This translates into an increase in the quality of life of patients and substantially reduces the resources that governments allocate to the public health system for their care. In general, the diagnostic criteria for CVD established by WHO/WHF include anamnesis, electrocardiogram, and clinical laboratory tests (Rapaport et al., 1979; World Heart Federation, 2022). Nowadays, the most requested clinical laboratory tests consider the determination of biomarkers that are directly related to myocardial damage. The most used biomarkers include procalcitonin (PCT), creatine kinase MB (CK-MB), natriuretic-type B pro-peptide (pro-BNP), cardiac troponin T (cTnT) and cardiac troponin I (cTnI) (Aydin et al., 2019; Tilea et al., 2021).

cTnI is a protein isoform that forms, together with cTnT and cardiac troponin C, the three polypeptide subunits of the troponin complex. cTnI is involved in the regulation mechanism of cardiac muscle contraction (Marston and Zamora, 2020). Its release into the bloodstream occurs simultaneously with cTnT in the presence of acute or subacute myocardial necrosis (Wen, 2011; Katrukha and Katrukha, 2021). However, cTnI is more differentiated from its isoform found in skeletal muscle, contrary to what happens between cTnT and its skeletal isoform (Gaze and Collinson, 2008; Yang et al., 2009; Marston and Zamora, 2020). Since its discovery in 1963, cTnI has been studied mainly in AMI, so that for this condition, the release kinetics (RK) of the biomarker has been well characterized (Ebashi, 1963; Bertinchant et al., 1996). Considering the RK of cTnI, this biomarker can be used to predict the recurrence of AMI in patients who have previously presented this event (Laugaudin et al., 2016). Also, the early diagnosis of AMI is possible considering measurements of cTnI concentrations at 0 h/1 h and 0 h/2 h (Collet et al., 2021). However, both assertions will be subject to the patient clinical history, ECG findings, monitoring, and disease control (Thygesen et al.*,* 2018). Due to its cardiospecificity, cTnI has demonstrated its functionality in the differential diagnosis, where there are different phases of myocardial ischemia associated with other cardiac conditions such as myocarditis, heart failure, ischemic heart disease, and stable angina, especially in relapsed patients (Korff et al., 2006; Januzzi et al., 2019; Tandon et al., 2019; Chaulin, 2021a; Chaulin, 2021b).

Diagnostic tests for cTnI are divided depending on the test sensibility, into conventional (from 0.005 ng/ml) and high or ultra-high sensitivity (below 0.005 ng/ml) immunoassays. This value is the reference cTnI concentration for healthy people (Keller et al., 2009; Januzzi et al., 2019). These tests can be carried out in centralized laboratories, with an average result output of one or more days, or with Point-of-Care diagnostic tests (POCT), with results between 5 and 15 min after sampling (Luppa et al., 2011; Abel, 2015; Florkowski et al., 2017). Currently, POCT present limitations when compared to centralized laboratory tests, such as lower analytical sensitivity, low precision and in some cases requires trained personnel for their use; however, they are a growing alternative for the disease management and control where timely and early diagnosis is vital, such as in CVD or diseases that can lead to the development of pandemics/epidemics (Bissonnette and Bergeron, 2010; Ferreira et al., 2018; Kumar et al., 2022). According to the POCT market report of the BCC Research (Business Communications Company), a compound annual growth rate (CAGR) of 11.9% is projected for the 2021-2026 period, which represents about 43.5 billion dollars (BCC Research, 2022). However, the most recent Markets and Markets report foresees a CAGR of 10.8% for the 2022-2027 period, which is equivalent to 72 billion dollars, an increase driven by the current SARS-CoV-2 virus pandemic (Cucinotta and Vanelli, 2020; Markets and Markets, 2022). This is due to the high demand for tests that allow rapid diagnosis without compromising its basic qualities of accuracy, sensitivity, speed, portability, and ease of use (Nichols et al., 2020). In this context, the POCT industry represents a new era in rapid disease diagnosis with applications in close-to-patient environments (hospitals, outpatient clinics and home care settings) but also with the possibility of being adapted to centralized clinical laboratories (Rajan and Glorikian, 2009; Nayak et al., 2017).

With respect to the impact of the current Coronavirus pandemic, recent studies have confirmed that the pre-existence of CVD potentiates the risk of being infected by COVID-19 and that people affected by this disease can be susceptible to develop cardiac complications (Aboughdir et al., 2020; Nishiga et al., 2020; Lippi et al., 2021). It has been reported that COVID-19 triggers an increase in the levels of blood biomarkers of cardiovascular diseases, particularly cTnI (Lippi et al., 2020; Shoar et al., 2020; Toraih et al., 2020; Ali et al., 2021; Haji et al., 2022), significantly reducing the life expectancy of the patient. Thus, this concrete example demonstrates the great importance of a rapid screening of cTnI and the need of implementing tools that provide reliable quantitative and multiplexed information of this biomarker (Wen et al., 2020; Orlov et al., 2022).The quantification of cTnI has been addressed mainly by immunoassay methods in sandwich format using different techniques such as surface plasmon resonance, fluorescence, electrochemistry, and surface enhanced Raman spectroscopy (SERS) (Campu et al., 2022). SERS stands out as a promising candidate for the development of point-of-care quantitative analysis of biochemical markers, due to its particular features such as the high sensitivity and possibility to perform simultaneous multiplexed measurements in different types of samples (dry and liquid samples), as well as minimal sample preparation (Huang, et al., 2019). In addition, modern Raman equipment have the flexibility to be adapted to a variety of technologies like optical fiber and microfluidics without making its operation complex (Marks et al., 2017; Quarin and Strobbia, 2021; Guo et al., 2022). In this way, emerging trends in the use of POCT devices pushed by the modern clinical practice needs, such as remote or drive-through testing, stand-alone platforms, multiplex detection in real time looks more real today. In this review, we discuss the advances in SERS-based strategies for cTnI detection published from 2016 to 2022, emphasizing parameters such as sample volume, linearity, interferents, and immunoreaction time that allow the recognition of POCT. A section of cardiovascular and non-cardiovascular diseases with cTnI release, including their patterns of release as a function of time or the cut-off value found in blood matrix is discussed. The above information was correlated with the detection range of the analyzed SERS-based sandwich ELISA immunoassays, disclosing their utility beyond the commonly addressed AMI diagnostic. Finally, point-of-care testing, the use of SERS as a POCT technique and some aspects of the biochemistry of cTnI are discussed.

## 2 Point-of-care testing

**FIGURE 1:**
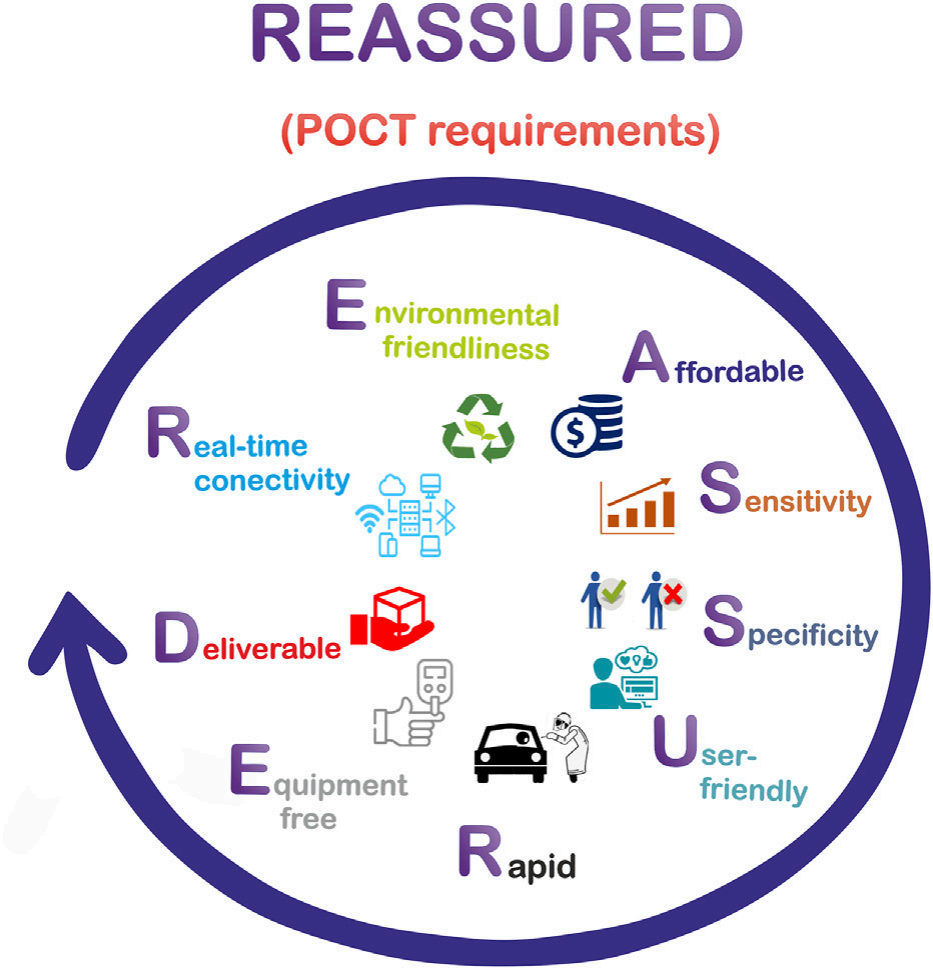
Meaning of REASSURED acronym.

POCT, near-patient testing (NPT), bedside testing (SBT), off-site testing (OT), and physician office laboratory (POL) are tests performed near or at the site of a patient with the result leading to a possible change in patient care (International Organization for Standardization, 2022). The first and most basic point-of-care (POC) test was published by Kohn et al. (1957), and consisted in an enzyme dipstick used for detecting glucose in urine (Kohn et al., 1957). To the late 1980s, this new technology was available in real environments to test glucose at the Massachusetts General Hospital in Boston (Goodson et al., 1986). However, at that moment, the introduction of POCT was precipitate, the technology was immature, and the devices present significant issues related to their analytical performance. In addition, the available menu of POCT was limited and the price per unit was higher than tests offered in centralized laboratories, which slowed down its use and development (Lee-Lewandrowski et al., 1994; Bailey et al., 1997; Baer, 1998). During the 1990s, few POCT tests were used, such as fecal immunochemical tests and urine dipstick tests (Uchida et al., 1990; Pritchard and Levernier, 1991). In 1992 Lewandrowski et al. again motivated the use of POCT by successfully implementing the first capillary blood glucose meter in hospital settings (Lewandrowski et al., 1992). Since that day on, the demand of POCT began to increase constantly and experienced an acceleration in the current COVID-19 pandemic, where POCT is becoming the protagonist in controlling and achieving early detection and prompt treatment (Yi et al., 2021; Kumar et al., 2022).The ideal requirements for a POCT system coincide with the criteria established by the World Health Organization Special Programme for Research and Training in Tropical Diseases (WHO/TDR) for the ideal diagnostic test that could be applied at all levels of the health care system, for clinical disease management of infectious tropical diseases and sexually transmitted infections. These criteria are abbreviated by the famous acronym ASSURED, as illustrates in Figure 1 (Otoo and Schlappi, 2022). R refers to real-time connectivity (results are added to an electronic medical record); E refers to the ease of specimen collection (does not require a venipuncture, so it does not require a health professional to perform it); A means affordability, S means sensitivity (it should avoid false negatives in diagnostics for detection purposes; in case of discrepancies, a second and even a third test is used as a tiebreaker); S refers to specificity (the specificity achieved in POCT diagnoses should be compared to those of laboratory tests); U refers to user-friendly (the diagnostic tests should be performed in 2 or 3 steps, requiring minimal personnel training or even without prior knowledge); R is related to a rapid test (the time for results emission must be between 15 and 60 min after the sample collection); E refers to equipment-free (the test must not use any equipment or must be operated on small portable devices), and D to deliverability refers to deliverable to end-users (involves the logistics related to the purpose of selecting, acquiring, shipping, storing, distributing, and delivering a new technology to end-users). These criteria accumulate as technology advances; for example, self-monitoring, syndromic diagnosis and environmentally friendly devices are now considered (Dima, 2021; Kim et al., 2021; Ongaro et al., 2022).

## 3 Surface enhanced Raman spectroscopy as a point of care technique

SERS is based in the enhancement of the Raman scattering phenomenon of a molecule when it is adsorbed typically on a plasmonic nanostructure, but also on dielectric nanostructures (SERS based on non-plasmonic substrates) (Alessandri and Lombardi, 2020; Barbillon, 2020; Cong et al., 2020). The detection of a single molecule by SERS was proved for the first time in 1997, using organic dye molecules on silver colloidal nanoparticles (Kneipp et al., 1997; Chalabi et al., 2014). From that day on, this achievement has been reproduced with other molecules and other plasmonic substrates, improving substantially the generic strategies (Mosier-Boss, 2017; Mao et al., 2018; Almehmadi et al., 2019; William et al., 2019). Due to the astonishing sensitivity obtained by SERS, the technique is being well accepted in the field of analytical chemistry.

The sensitivity of the SERS technique depends on the enhancement of the Raman scattering of the adsorbate, which is evaluated in terms of the enhancement factor (EF) (Le Ru et al., 2008). Two mechanisms acting synergistically contribute to this enhancement: the electromagnetic (EM) and chemical (CM) mechanisms (Kambhampati et al., 1998). There is a plethora of articles and book chapters (Le Ru and Etchegoin, 2009; Li et al., 2014; Wang J. et al., 2021) explaining in detail the basis of these effects, and the goal of this review is not to describe their fundamentals. However, it is important to consider that the EM mechanism is the distinctive mark of plasmonic nanostructures, mainly silver and gold, and, with an EF value of up to ∼10^6^, it is considered the major contributor to the Raman signal enhancement (Le Ru and Etchegoin, 2009; Li et al., 2014).

A minor contribution to the total Raman enhancement comes from the CM. Charge transfer (CT) between the metal or semiconductor and the adsorbed molecule, or *vice versa*, is one of the most attractive routes of CM contribution. This occurs during the formation of surface complexes, triggered by photoinduction or mediated by specifically adsorbed species (Yang et al., 2019; Pan et al., 2021; Wang L. et al., 2021). CT contributes with a signal amplification of 10^3^, while static charge transfer can contribute with amplifications as subtle as 10 times or less or as significant as 100 times (Cong et al., 2020). Special contributions to the chemical enhancement mechanism of SERS are the surface-enhanced resonance Raman scattering (SERRS) and the plasmon-like resonant mechanism (PL-SERS). The former occurs when the excitation laser is tuned into or near an electronic transition of the molecule that is attached on a SERS substrate. Under this condition an improvement of the conventional resonance Raman scattering of the molecule is induced by its proximity to the substrate (López-Castaños et al., 2020; Moldovan et al., 2022). The PL-SERS arises from the coupling of certain normal modes of the adsorbate with internal resonant excitations of small metal clusters (Román-Pérez et al., 2014). However, the CM is in exploration and faces difficulties to contrast experimental results with theoretical predictions, due in part to the coupling, simultaneity and overlap of the contributions under ordinary conditions. Likewise, the relationship between EM and CM is still not clear, but ceaseless efforts are being made to clarify these questions to open the possibility of a predictive design and manipulation of analytical systems in the near future (Mueller et al., 2016; Kim et al., 2019; Mueller and Reich, 2019; Neuman et al., 2019).

In addition to its great sensitivity, another appealing of SERS to be used as an analytical tool is its capacity for rapid and point-of-care (POC) tests. Portability, autonomy, simplicity of use, fingerprint information, speed, little or no sample preparation, and the possibility of evaluation of samples with different moisture content or in different states of matter, are attributes that come from the instrumentation and from the Raman technique itself. It also opens the possibility of conducting different types of analysis (qualitative/quantitative, multiplex/monoplex, dry/wet, label free/label based and independent/simultaneous) by changing the strategies and the analysis system (Xu et al., 2019; Wang T. et al., 2021; Mousavi et al., 2022). Despite the merits of SERS that predict its excellent performance in POCT, there are limitations that hinder its real-life application in this area: 1) elevated cost of the Raman spectrometer compared to routine analytical instruments such as fluorescence and UV/vis spectrometers; 2) the need for lasers of different wavelengths to analyze a wide range of analytes, which low the technique throughput; 3) poor reproducibility and repeatability of experimental data. When using colloidal metal nanoparticles, these problems come from uncontrolled movement of plasmonic particles in the fluid (Brownian motion). In the case of the SERS substrates, problems arise from: 1) the oxidation of the metallic nanoparticles when classic plasmonic substrates are used (naked and monometallic nanoparticles) or 2) the difficulty to replicate the same hot-spot pattern on the substrate surface; 4) inefficient approaches for pretreatment, preprocessing and processing spectral data that decrease the technique accuracy and precision. To bring this technology to real-life POCT applications, it is necessary to address the above drawbacks from different disciplines. For instance, a better understanding of the SERS driving mechanisms will allow customizing each analytical system. With this information available, optimization can be carried out not only by following the SERS substrate performance but also by considering the chemical nature of the adsorbate and its properties when in contact with surfaces and light. On the other hand, the required sensitivity depends on the analyte; therefore, it is possible to establish a balance between benefit (signal intensity) and cost (laser type) by considering the contribution of both EM and CM mechanisms. Through this means, it will be possible to modulate the Raman signal, considering the use of cheaper and efficient laser excitations. Finally, a deeper comprehension of SERS fundamentals must be combined with tailored data analysis pipelines (pretreatment, preprocessing and processing spectral data) for the accurate analysis of Raman data. In this sense, the utilization of advanced machine learning algorithms, such as neural networks (NNs) are emerging.

## 4 cTnI in cardiovascular and non-cardiovascular diseases

cTnI is released under the myocardial injury process mainly in the bloodstream, although its presence has been reported in body fluids such as saliva, sweat and urine (Tadepalli et al., 2015; Chekin et al., 2018; Chaulin et al., 2020; Chen et al., 2020). Its liberation often occurs as a response to cellular maintenance processes of the cardiovascular system, such as cell turnover, cardiovascular homeostasis, and cellular stress (Koerbin et al., 2012; Chaulin, 2021c). These processes are non-pathological, and are the result of biochemical mechanisms that include the regeneration of myocardiocytes, the release of fragments from proteolytic degradation of troponins, the transport of cTnI in membrane vesicles, the increase in the permeability of myocardiocyte membranes, and programmed cell death (apoptosis, autophagy and necroptosis) (Schwartz et al., 1984; Ross and Borg, 2001; Berghe et al., 2009; White, 2011; Moreno, 2016; Eschenhagen et al., 2017; Chaulin, 2021c). However, when cTnI release occurs as a consequence of pathological processes, cardiac and non-cardiac diseases can be differentiated (Chaulin, 2021a). Table 1 summarizes some of the most studied cardiac and non-cardiac diseases associated with the release of cTnI together with information about their release mechanism.

**TABLE 1:** Cardiac and non-cardiac diseases with cTnI release and the proposed mechanisms.

Cardiac diseases	Proposed mechanism of cTnI release	References
Acute myocardial infarction	• Intraluminal platelet aggregation resulting in partial or complete vascular occlusion.	Heusch et al. (2009); O’Gara et al. (2013); Amsterdam et al. (2014); Heusch, (2016); Thygesen et al. (2018)
	• Release of platelet microaggregates resulting in microembolization of small vessels that cause localized ischemia and infarction.	
	• Rupture or ulcers in the atherosclerotic plaque, generating an intraluminal thrombus with manifestations of acute myocardial ischemia.	
Cerebrovascular accidents	• Secondary cardioembolic cerebral ischemia related to primary myocardial damage.	Christensen et al. (2004); Barber et al. (2007); Tanindi and Cemri, 2011)
	• Myocardial damage caused by sympathoadrenal activation.	
	• Acute brain injury caused by an increase in plasma catecholamines.	
Unstable angina	The same as in acute myocardial infarction.	Defilippi and Mills, (2021)
Acute and chronic heart failure	• Subendocardial ischemia leading to myocyte necrosis.	Levine et al. (1990); Sato et al. (2004); Hessel et al. (2008); Kociol et al. (2010); Tanindi and Cemri, (2011); Januzzi et al. (2012)
	• Cardiomyocyte damage by inflammatory cytokines or oxidative stress.	
	• Hibernating or apoptotic myocardium.	
	• Increased plasma membrane permeability.	
	• Leakage from the cytosolic pool of cTnI.	
Acute myocarditis	Inflammation of the myocardium associated with the direct cytotoxic effect of infectious agents (viruses, bacteria), toxins, and autoantibodies on cardiomyocytes.	Smith et al. (1997); Yozgat et al. (2020)
Endocarditis/Pericarditis	Inflammation of endocardial and epicardial cells is often accompanied by the release of cTnI.	Brandt et al. (2001); Watkin et al. (2004)
Aortic valve disease	Myocardial strain due to an increase in left ventricular wall thickness-pressure and pulmonary artery systolic pressure.	Nunes et al. (2003)
Hypertrophic cardiomyopathy	• Cardiomyocyte apoptosis due to thickening (hypertrophy) of the myocardium.	Gommans et al. (2013); Osmanska et al. (2020)
	• Imbalance between oxygen demand and supply caused by a sharp increase in demand from hypertrophied myocytes, leading to fibrosis.	
Tachycardia/Tachyarrhythmia	Temporary myocyte damage due to increased myocardial demand of oxygen and nutrients as a result of shortened diastole.	Redfearn et al. (2005); Patanè et al. (2009); González-Del-Hoyo et al. (2019)
Arterial hypertension	• Activation of the apoptotic processes of cardiomyocytes, caused by their stretching, or increased activity of the adrenergic system.	Chaulin, (2021d); Chaulin and Duplyakov, (2021)
	• Activation of proteolytic degradation processes in cardiomyocytes.	
	• Increased membrane permeability of cardiomyocytes due to myocardial overload or damage of membrane components.	
	• Negative effect of blood pressure on elimination processes in the blood serum.	
	• Stress/tension of the left ventricle wall.	
	• Myocardial hypertrophy.	
Takotsubo syndrome	Direct myocardial toxicity provoked by excess of circulating catecholamines.	Rahman and Broadley, (2014); Kai and Bertil (2017)

Non-Cardiac diseases	Proposed mechanism of cTnI release	References
Chronic obstructive pulmonary disease (COPD)	Pulmonary hypertension, hypoxia, and hypercapnia contribute to myocardial damage and death.	Baillard et al. (2003); Martins et al. (2009)
Acute pulmonary embolism	• Increased cTnI is probably associated with the release from the cytosolic pool across the membrane of the right ventricular cardiomyocytes.	Torbicki et al. (2008); Lippi et al. (2019); Chaulin, (2021b)
	• Vasoconstriction of the small branches of the coronary arteries, and impaired hemoperfusion lead to ischemia of myocardiocytes.	
	• Cardiomyocyte necrosis may also occur due to increased oxygen demand.	
Strenuous exercise^a^	• Transient ischemia due to an imbalance of oxygen demand in cardiomyocytes (high metabolic activity to maintain contractile activity).	Lippi et al. (2011); Lavie et al. (2015); Paana et al. (2019); Chaulin, (2021a)
	• Excessive myocardial hypertrophy.	
	• Cardiomyocyte death through small-scale (subclinical) necrotic and apoptotic processes.	
Diabetes mellitus	• Myocardial ischemia caused by an imbalance between oxygen demand and supply.	Grundy et al. (1999); Vazzana et al. (2011); Eubanks et al. (2012)
	• Myocardial hypertrophy caused by increased arterial wall stiffness.	
	• Decrease in the clearance of cTnI molecules from the blood serum due to a decrease in the glomerular filtration rate in chronic kidney disease.	
Infiltrative diseases (rhabdomyolysis, cardiac amyloidosis, septicemia)	• *Rhabdomyolysis*: Cardiotoxicity due to the release of free radicals, circulating cytokines and ion fluxes. Muscle damage by activated neutrophils that trigger both local and systemic inflammatory response.	Löfberg et al. (1996); Cantwell et al. (2002); Punukollu et al. (2004); Bessière et al. (2013); Zimmerman and Shen, (2013); Perfetto et al. (2014); Wilhelm et al. (2014); Sheyin et al. (2015)
	• *Cardiac Amyloidosis*: Proinflammatory and toxic effects of amyloid protein leading to inflammation of myocardial cells, which could cause ischemia and the subsequent release of cTnI.	
	• *Septicemia (sepsis)*: Myocardial ischemia produced by inflammatory mediators (tumor necrosis factor alpha (TNF-ɑ), interleukins (IL-1, IL-6), and bacterial exo- and endo-toxins.	
Severe pulmonary hypertension	• Elevated pulmonary vascular resistance leads to increased right ventricular ischemia and necrosis.	Heresi et al. (2012); Vélez-Martínez et al. (2013); Chaulin, 2021b)
	• The increase in C-reactive protein triggers the inflammatory mechanism involved in the increase of cTnI levels.	
Acute and chronic kidney failure	• Increased molecules in the blood circulation due to decreased glomerular filtration rate.	Ziebig et al. (2003); Khan et al. (2005); Defilippi and Herzog, (2017); Chaulin, (2021a); Hong et al. (Forthcoming 2022)
	• Myocardial volume overload and its hypertrophy.	
	• Direct damaging effect of the toxic products of nitrogen metabolism with the consequent damage and death of cardiomyocytes.	
COVID-19 (early heart damage)	• Increased systemic inflammatory response (high levels of lymphocyte count, CRP, and LDH).	Shi et al. (2020); Zhou et al. (2020); Lin et al. (2022)
	• Lung compromise.	
	• High viral response.	
	• Low oxygen saturation levels.	

COVID-19, Coronavirus disease 2019; CRP, C-reactive protein; LDH, Lactate dehydrogenase.

^a^ Although this is not a disease, it is considered here because of the associated release of cTnI.

**FIGURE 2:**
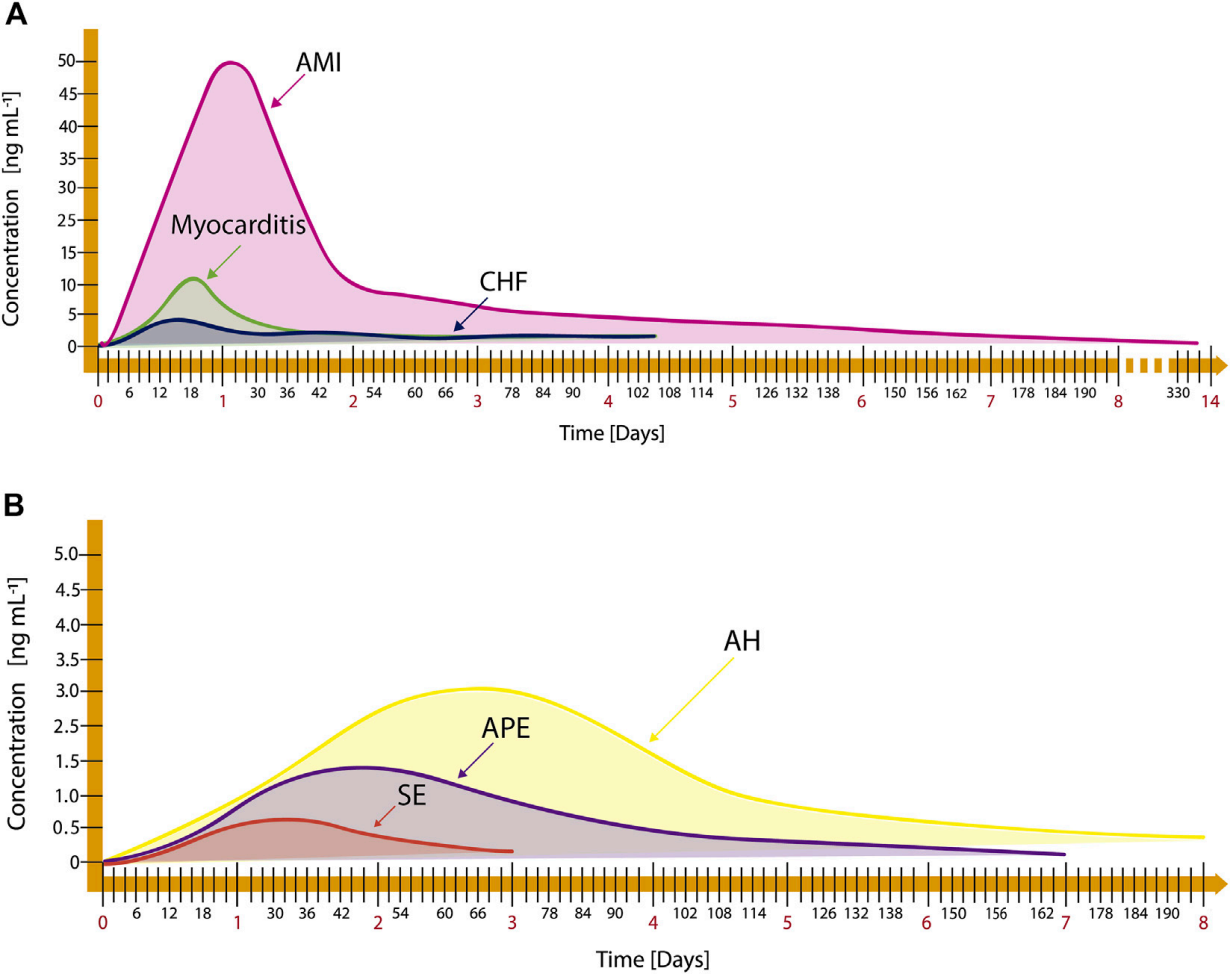
Release kinetics of cardiac and non-cardiac diseases: **(A)** AMI, myocarditis, and Chronic Heart Failure (CHF), adapted from Mahajan and Jarolim (2011); Abdolrahim et al. (2015), and **(B)** Arterial Hypertension (AH), Acute Pulmonary Embolism (APE), and Strenuous Exercise (SE), adapted from Douketis et al. (2002); Taddei et al. (2011); Brzezinski et al. (2019).

To establish a diagnosis of the diseases shown in Table 1, the cTnI cut-off values should be considered (see Table 2). The disease is present when the cTnI concentration is above this value. Likewise, the available RK values of cTnI for diseases of Table 1 are presented in Figure 2. The knowledge of these values facilitates an early diagnosis, monitoring, and prognosis of diseases.

**TABLE 2:** cTnI cut-off values for the cardiac and non-cardiac diseases presented in Table 1.

Disease	Cut-off (ng/ml)	
	Cardiac diseases	
Acute myocardial infarction	0.042	Kampmann et al. (2022)
Acute heart failure	> 1.0	Peacock et al. (2008)
Acute myocarditis	0.173–2.581	Liu Y. et al. (2020)
Endocarditis	0.01–5.68	Mellanby et al. (2009)
Pericarditis	0.01–0.486	Mellanby et al. (2009)
Aortic valve disease	0.68–2.59	Van Geene et al. (2010)
Cardiomyopathy	2.11–2.581	Pretorius et al. (2014)
Tachycardia	4.1	Bertsch et al. (1997)
Tachyarrhythmia	0.571	Mariathas et al. (2018)
Unstable angina	0.126–0.148	Jordanova et al. (2005)
Arterial hypertension	1.14	Yang, Xing and Li, (2021)
Stroke	0.0887	Pop et al. (2021)
Takotsubo syndrome	0.115	Dagrenat et al. (2020)
	Non-cardiac diseases	
COPD	0.031	Nilsson et al. (2020)
Acute pulmonary embolism	0.06	Kucher et al. (2003)
Acute kidney failure	0.02–0.24	Song et al. (2012)
Chronic kidney failure	0.0145–0.054	Ziebig et al. (2003)
Strenuous exercise	0.014–27.2	Eijsvogels et al. (2014)
Diabetes	0.05–0.40	Eubanks et al. (2012)
Rhabdomyolysis	> 0.6	Punukollu et al. (2004)
Amyloidosis	0–24	Dispenzieri et al. (2003)
Septicemia (sepsis)	1.02	Croal et al. (2006)
Severe pulmonary hypertension	0.01	Heresi et al. (2012)
COVID-19/Early cardiac damage	0.006–0.0156	Lin et al. (2022)
	Healthy individual	
	0.005	Januzzi et al. (2019)

## 5 cTnI biochemistry

In 1963, a new protein constituent of the cardiac myofibrillar apparatus was discovered (Ebashi, 1963). However, it was not until 1971 that this protein was identified and named the troponin complex integrated of three components: troponin T (TnT), troponin C (TnC) and troponin I (TnI) (Greaser and Gergely, 1971). For the first time in 1978, and as result of deeper studies regarding myocardial cell death, cardiac and skeletal muscle troponin I isoforms were identified (Cummins and Perry, 1978). Later, in 1983 the cardiac isoform of troponin T and its skeletal isoform were also identified for first time (Toyota and Shimada, 1983). In 1987, it was found that the isoform of cardiac muscle troponin I is more differentiated than its skeletal isoform, and that both present a cross-reactivity of about 1%–2%. On the other hand, the troponin T isoforms present a cross-reactivity of up to 4% (Cummins and Perry, 1978; Cummins et al., 1987; Kobayashi et al., 1992; Bodor et al., 1997). Since 2000, the use of cTnI has been recommended as a preferential cardiospecific biomarker for the evaluation of patients with possible CVD (Antman et al., 2000).

**FIGURE 3:**
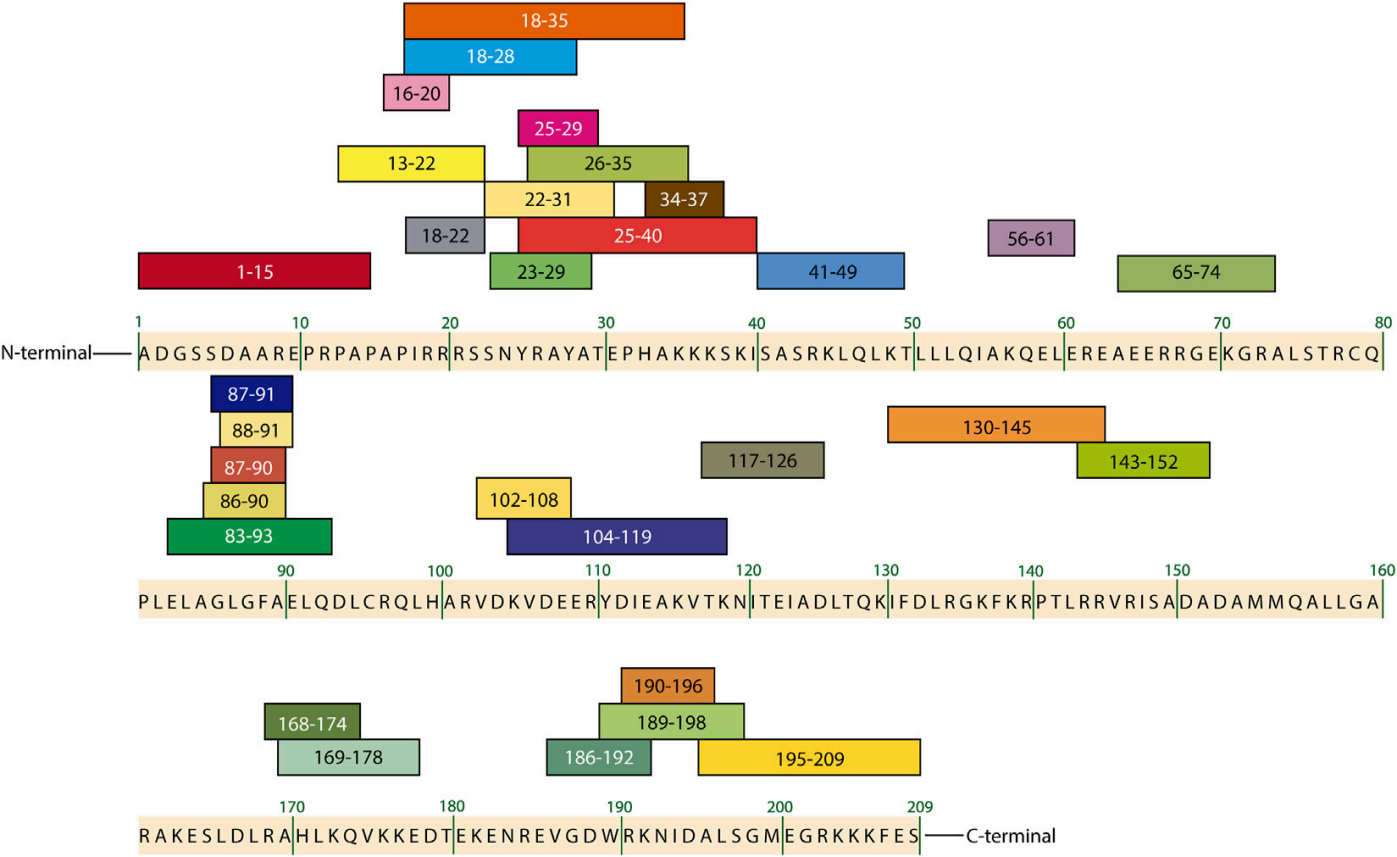
Epitopic map of cTnI containing the amino acid sequences of the most important external and internal epitopes adapted from HyTest (2022) and Filatov et al. (1998).

Cardiac troponin I in human is considered a highly specific cardiac biomarker. It is present in cardiac muscle tissue as an isoform of 209 amino acid residues, with a molecular weight of approximately 24 kDa and an experimental isoelectric point between 6.8 and 8.5 (Filatov et al., 1999; Gomes et al., 2002; Peronnet et al., 2007; Apple et al., 2012). Three different isoforms of troponin I (TnI) can be found in humans: the cardiospecific troponin I isoform (cTnI) and two isoforms that are expressed in skeletal muscle, named slow-twitch troponin I (ssTnI) and fast-twitch troponin I (fsTnI) (Hunkeler et al., 1991; Gaze and Collinson, 2008; Sheng and Jin, 2016). The three isoforms are highly homologous: the sequence identity of cTnI with ssTnI is approximately 52% and with fsTnI is 46% (HyTest, 2022). Despite of this, the cTnI isoform is well-differentiated from the skeletal isoforms because it presents an N-terminal region between amino acid residues 1-32, considered the cardiospecific region (Al-Hillawi et al., 1995; Keane et al., 1997; Larue et al., 1998). Once a cardiac event occurs, the great majority >95% of the blood cTnI has been found in the detectable dominant forms such as the non-covalent ternary cTnI-TC complex (TIC complex), the binary cTnI-C complex (IC complex), and the cTnI-T binary complex (IT complex). The free form is present in a minor quantity (∼5%) (Katrukha et al., 1997; Apple et al., 2012). On the other hand, the cTnI molecule presents highly antigenic regions that correspond to the amino and carboxyl-terminal extremes and internal antigenic sequences (Ferrieres et al., 1998). Figure 3 shows the epitopic map of cTnI that contains the external and internal epitopes reported so far (Filatov et al., 1998; HyTest, 2022).

In blood, these cTnI antigenic sequences generate an immune response in the body by inducing the production of antibodies that selectively recognize the cTnI epitopes. This chemical interaction can be interrupted by other substances called interferents. Endogenous interferents for cTnI are bilirubin, albumin, D-dimer, immunoglobulin G (IgG), as well as fibrin strands. Examples of exogenous interferents are certain medications and drugs such as heparin, EDTA, and clopidrogrel, warfarin, digoxin (Katrukha et al., 1999; Eriksson et al., 2003; Tate and Ward, 2004; Dimeski, 2008). On the other hand, taking the advantage of the same antigen-antibody interaction, the large-scale production of a variety of antibodies against cTnI has been possible, polyclonal (pAbs) and monoclonal (mAbs) antibodies produced by traditional hybridoma-based technologies and recombinant ones (rAbs) generated *in vitro* by using genetics (Hyytiä, 2015; O’Kennedy and Murphy, 2017; HyTest, 2022), which have been used in the development of analytical strategies to detect cTnI.

## 6 Label free and label based determination of cTnI by surface enhanced Raman spectroscopy

**FIGURE 4:**
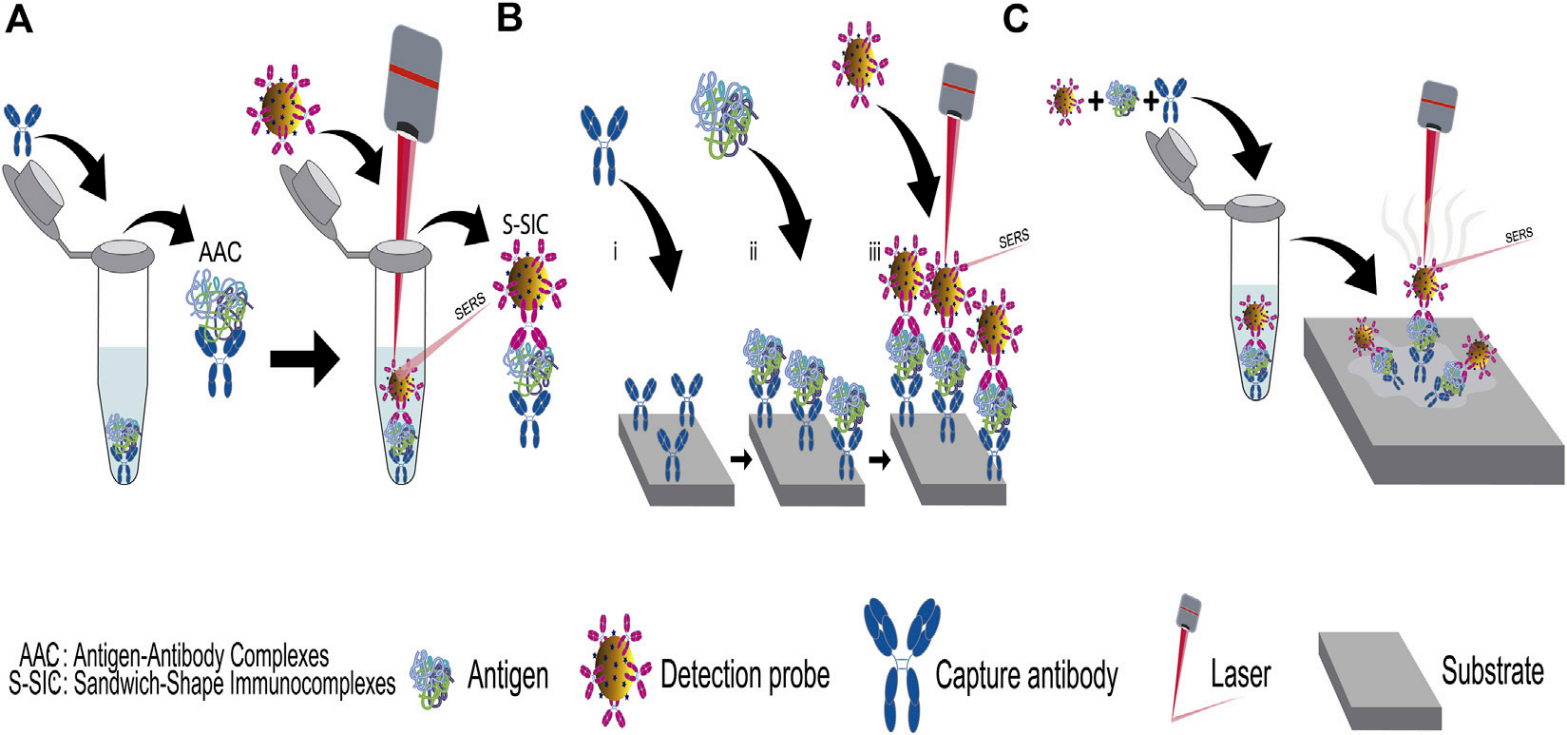
SERS measurement routes. **(A)** in solution, **(B,C)** on substrates.

There are two approaches for the SERS detection, label free (LF) and label based (LB) (Chisanga et al., 2019; Pilot et al., 2019). LF consists in a direct detection that is achieved by following the signal of a Raman-active analyte around a plasmonic metal nanoparticle. When the analyte is not close enough to the metal surface to be benefited by the electromagnetic field around the nanoparticle, presents a low Raman cross-section, exhibits Raman scattering competing phenomena (fluorescence) or simply the obtained SERS signal does not meet the desirable analytical requirements, the LB detection is the best option. In the LB approach the Raman signal of a metabolite, reaction product or Raman reporter molecules (RRMs, molecules that possess strong Raman activity) can be tracked in order to indirectly detect the analyte. The LB route is the most efficient to detect biomarkers in biological samples. Regardless of the selected approach, one the most popular diagnostic strategy is the use of sandwich enzyme-linked immunosorbent assay (ELISA) (Liu R. et al., 2020). SERS-based sandwich ELISA comprises the use of capture antibodies that can be free or attached on solid phases (named capture substrates). From their interaction with the antigen, the antigen-antibody complexes (AAC) are formed. On the other hand, the detection or signal probes in solution are constituted of a second antibody immobilized on a SERS-active nanostructure, mainly gold nanoparticles. The intrinsic Raman signal of biomarkers are weak with large fluorescence emission, whence RRMs are typically added onto the detection probes to achieve the quantification of the biomolecule by the LB approach. By the interaction of the detection probes with the AAC, the sandwich-shape immunocomplexes (S-SIC) are formed (Smolsky et al., 2017). Although antibodies are the generic biomarker-capture reagents used in the ELISA immunoassay method, they can be substituted by aptamers, a more efficient oligonucleotide-based reagent (Thiviyanathan and Gorenstein, 2012; Kimoto et al., 2019; Bognár and Gyurcsányi, 2020; Wang X. et al., 2021; Majdinasab et al., 2022). Once the immunoassay is completed, the Raman experiments can be performed either directly in the solution where the S-SIC were formed or in a dry way on the capture substrate where the immunoassay was driven (see Figures 4A,B). Another means of conducting the Raman experiments is by forming a thin film of the S-SIC from evaporation of the immunoassay solution on a substrate, commonly a glass slide, as shown in Figure 4C.

**FIGURE 5:**
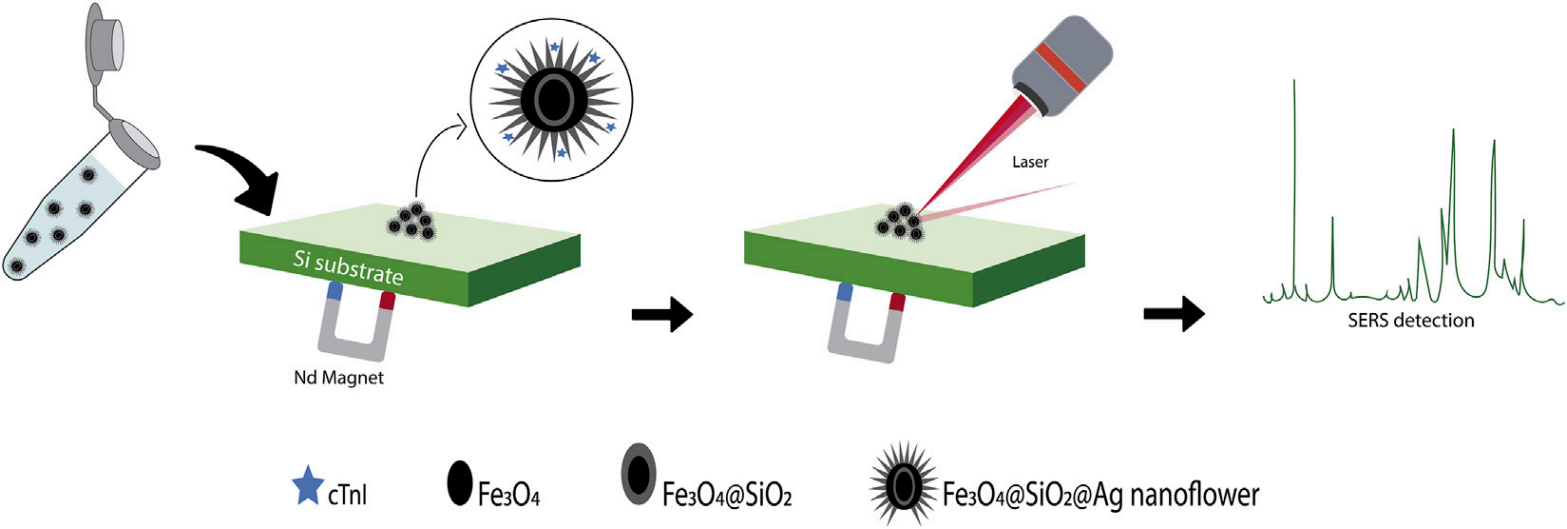
Schematic representation of the aptasensor based on a flower-shaped silver magnetic nanocomposite for the label-free detection of cTnI by SERS, adapted from Alves et al. (2020).

Detection of cTnI by SERS has been addressed with both LF and LB routes, the latter being mainly based on the use of RRMs. In the time frame considered in this review (2016-2022), the only article where the immunoassay strategy for detection of cTnI was carried out through the LF route was that published by Alves et al. (2020). They used flower-shaped Fe3O4@SiO2@Ag nanoparticles functionalized with cTnI binding-5′ thiol modified aptamer (Figure 5) as detection probes. After 10 s of interaction of the capture probes with the cTnI containing solution, the SERS measurements were carried. For this, 10 μl of the cTnI-detection probe suspension was dropped onto a Si substrate. The sample was subsequently concentrated and evaporated by placing a magnet under the Si substrate. The cTnI was detectable at a concentration as low as 10 ng/ml by a direct monitoring of the cTnI signal at 944 cm^−1^ related to the C–C stretching vibration. No interference studies or real sample analysis were reported in this work.

The available SERS approaches for the detection of cTnI using the LB route are immunoassays carried out either in solution or on substrates and include the measuring aspects shown in Figure 4. From this information, Table 3 was elaborated to summarize some of the most relevant characteristics that a POC assay should exhibit. Cardiac and non-cardiac diseases that could be diagnosed with the developed devices are also considered in this table.

**TABLE 3:** Immunoassays for cTnI measurement taken from the literature published between the years 2016–2022.

LOD ng/mL	Linearity ng/mL	Tested matrix	Excitation laser (nm)	RRM/Band marker (cm^−1^)	Immunoreaction time	Specificity	Sample volume (μL)	Validated method	Diseases reported in Table 2 that can be diagnosed	References
0.005	0.01–1,000	SSM	633	MGITC/1,617	AAC: 120 min	IgG, PSA, CEA, GC	---	------	All, except: COVID-19/Early cardiac damage and healthy individuals	Fu et al. (2019)
					S-SIC: 60 min					
0.0098	-----	HS	-----	4MBA/1,075	Less than 30 min (37°C)	H-FABP, D-dimer, NT-proBNP, BSA	50	CLIA	-----	Hu et al. (2021)
0.0044	------	HS	-----	4-MP/1,073	Less than 30 min (37°C)	NT-proBNP, BSA, D-dimer	60	CLIA	-----	Hu et al. (2020)
0.0000229	0.0001–0.1	HS	785	TMB/1,608	AAC: 60 min	Myo, CRP, cTnT	100	commercial ELISA kit	Stroke, COPD, APE, CKD, SPH, COVID-19/Early cardiac damage and heathy individuals	Lee et al. (2022)
	S-SIC: 60 min									
	TMB product formation:10 min									
----	0.01–1.0	HS	638	4MBA/1,609	30 min (25°C)	IgG, LPL, AchE, HSA, CEA	20	------	All, except: endocarditis, AVD, cardiomyopathy, tachycardia, amyloidosis, sepsis, and healthy individuals	Lin et al. (2021b)
------	------	---	532	DTNB/1,336	30 min	-----	30	-----	-----	Garza and Cote (2017)
0.00316	0.01–100	WB	785	DTNB/1,333	AAC: 60 min	Myo, CEA, AFP, HSA, IgG	15	----	All, except: COVID-19/Early cardiac damage and healthy individuals	Wang J. et al. (2021)
	S-SIC: 60 min (37°C)									
7.6 × 10^−7^	1 × 10^−6^–1,000	HS	633	DTNB/1,323	AAC: 40 min	BSA, GC, GLU, IgG, IL-6	20	standard ELISA	All	Su et al. (2018)
	S-SIC: 40 min (37°C)									
0.0089	----	HS	632	MGITC/1,615	AAC: 60 min	IgG, HSA, BSA, Myo, CK	3	CLIA	-----	Cheng et al. (2019)
	S-SIC: 60 min									
0.0024	0.00024–2.4	HS	633	Cy5/1,580	A-APTC: 360 min	TnC, TnT, IgG, avidin	1,000	standard ELISA	AMI, tachyarrhythmia. UA, stroke, COPD, APE, AKD, CKD, diabetes, heathy individuals	Lee et al. (2020)
	S-SIC: 30 min (35°C)									
0.0055	0.01–100	HS	638	CBBG/1,621	70 min (25°C)	IgG, LPL, AchE, HSA, CEA	100	------	All, except: COVID-19/Early cardiac damage and healthy individuals	Lin, et al. (2021b)
0.0055	0.02–1.0	HS	785	DTNB/1,325	AAC: 60 min	---	25	CLIA	AMI, tachyarrhythmia. UA, Stroke, COPD, APE, AKD, CKD, diabetes	Wen et al. (2020)
	S-SIC: 60 min (4°C)									
0.00044	0.01–50	HS	785	NBA/592	15 min per T-line	---	100	CLIA	All, except: COVID-19/Early cardiac damage and healthy individuals	Zhang et al. (2018a)
0.00089	0.01–50	HS	785	NBA/592	7 min	---	100	CLIA	All, except: COVID-19/Early cardiac damage and healthy individuals	Zhang et al. (2018b)
0.09	0.09–50	HS	785	NBA/592	15 min	CRP, BNP, Myo, CK-MB	100	---	AHF, Cardiomyopathy, tachycardia, tachyarrhythmia	Bai et al. (2018)
	UA, AH, rhabdomyolysis, sepsis									
0.1	0–0.3	--	785	NBT/1,280–1,360	10 min	---	70	---	AMI, UA, Stroke, COPD, APE, AKD, CKD, SPH	Khlebtsov et al. (2020)
	COVID-19/Early cardiac damage and healthy individuals									
0.016	0–0.1	HS	638	MGITC/1,617	40 min	CRP, BNP, H-FABP	70	----	AMI, Stroke, COPD, CKD, SPH, COVID-19/Early cardiac damage and healthy individuals	Tu, Holderby et al. (2020)

LOD, limit of detection. SSM, serum substitute media. HS, human serum. WB, whole blood. RRM, Raman reporter molecule. MGITC, malachite green isothiocyanate. 4MBA, 4-mercaptobenzoic acid. 4-MP, 4-mercaptobenzonitrile. TMB, 3,3,5,5-tetramethylbenzidine. DTNB, 5,5-dithio-bis-(2-nitrobenzoic acid). Cy5, cyanine 5. CBBG, Coomassie brilliant blue G-250. NBA, Nile blue A. NBT, 1, 4-nitrobenzenthiole. AAC, antigen-antibody complexes. S-SIC, Sandwich-Shape Immunocomplexes. A-APTC, antigen-aptamer complexes. IgG, human immunoglobulin G. PSA, prostate specific antigen. CEA, carcino-embryonic antigen. GC: glucose. H-FABP: heart-type fatty acid-binding protein. NT-proBNP: N-terminal pro-brain natriuretic peptide. BSA: bovine serum albumin. Myo, myoglobin. CRP, C-reactive protein. cTnT, cardiac troponin T. LPL, human lipoprotein esterase. AchE, human acetylcholinesterase. HSA, human serum albumin. CK, creatine kinase. AFP, α-fetoprotein. GLU, glutathione. IL-6, Interleucina-6. CK-MB, kinase-MB isoenzyme. TnC, troponin C. TnT, troponin T. BNP, brain natriuretic peptide. CLIA, chemiluminescence immunoassay. ELISA, enzyme-linked immunosorbent assay. COVID-19, coronavirus disease 2019. CODP, Chronic obstructive pulmonary disease. APE, Acute pulmonary embolism. CKD, Chronic kidney disease. SPH, Severe pulmonary hypertension. AVD, Aortic valve disease. UA, Unstable angina. AKD, Acute kidney injury. AMI, acute myocardical infarction. AHF, Acute heart failure. AH, arterial hypertension.

### 6.1 Measurements in solution

**FIGURE 6:**
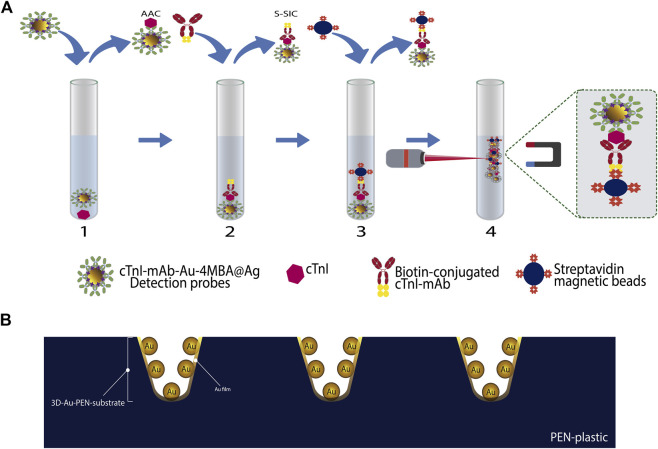
Schematic representation of different cTnI detection systems working in solution. **(A)** sandwich immunoassay, adapted from Hu et al. (2021) and **(B)** 3D AuNPs-PEN substrate, adapted from Lee et al. (2022).

Fu et al. (2019) developed a SERS-based sandwich ELISA immunoassay to detect cTnI. In this work, the capture substrate, antibody-functionalized magnetic beads in suspension, interacted with the sample that contained the cardiac biomarker to form the AAC. The S-SIC were formed once detection probes were added. These detection probes were composed by pAb-cTnI that were immobilized onto the surface of graphene oxide sheets (GO) modified with malachite green isothiocyanate (MGITC) functionalized gold nanoparticles (AuNPs), the system being labeled as pAb-MGITC-AuNPs@GO. Both procedures (AAC and S-SIC formation) involved incubation, recovery of the magnetic beads with an external magnetic field and washing steps. Finally, the S-SIC were redispersed in phosphate buffer solution (PBS) and the supernatant solution transferred to a capillary tube for Raman measurement. With this methodology, the increase in cTnI concentration increases the density of the signal probes on the surface of the magnetic beads, resulting in an enlargement of the SERS signal from the reporter molecule that allowed the biomarker quantification. Hu et al. (2021) reported a proposal performing the immunoassay and the Raman measurements in the same container. The detection probes consisted of a monoclonal antibody-conjugated on gold-silver core-shell nanoparticles with 4MBA encapsulated between the two metals (cTnI-mAb-Au@4MBA@Ag). The analysis process consisted of four steps as depicted in Figure 6A. In the first step, the interaction of the sample with the detection probes occurs. In the second step, biotin-conjugated cTnI-mAbs capture antibodies are added to form the double antibody sandwich complexes. The third step consists in the incorporation of streptavidin magnetic beads to achieve the specific reaction of streptavidin with biotin present on the capture probes. In the last step, after three washes with PBS solution, the magnetic beads are concentrated with the aid of a magnet and the Raman experiments conducted. The Raman intensity of the characteristic peak of the RRM showed a non-linear relationship with cTnI concentration in the range from 0 to 2.0 ng/ml. The spike recovery of the biomarker was between 85% and 120% in the presence of exogen interferents like heparin sodium, EDTA and trisodium citrate as well as endogen interferents such as bilirubin and hemoglobin (Hb). Using this quantification strategy, the same authors reported another work with the simultaneous detection of cTnI and H-FABP (Hu et al., 2020). The detection probe and the capture antibody to cTnI were the same, and to detect the H-FABP biomarker they added another RRM to the detection probes and used a capture antibody against H-FABP connected to biotin to participate in the immune response. The concentration of the two biomarkers was quantified by detecting the characteristic Raman peak intensities of their corresponding RRM. For cTnI, the values of validation parameters were similar to those obtained in the monoplex detection (see Table 3). Lee et al. (2022) developed a three-dimensional nanoreactor array as a SERS substrate that consisted in nanodimple arrays created on a polyethylene naphthalate (PEN) plastic film covered with AuNPs (3D-Au-PEN substrate, Figure 6B). The detection of cTnI was carried out in liquid-phase using the 3D SERS substrate in an 8-well plate configuration that were modified with anti-cTnI. As detection probes, AuNP were functionalized with HRP-conjugated anti-cTnI. The biomarker quantification was carried out after the S-SIC formation, tracking the activity of the HRP present in the detection probes, through the Raman signal of the TMB, which is the enzyme-substrate complex product.

### 6.2 Measurements on substrates

**FIGURE 7:**
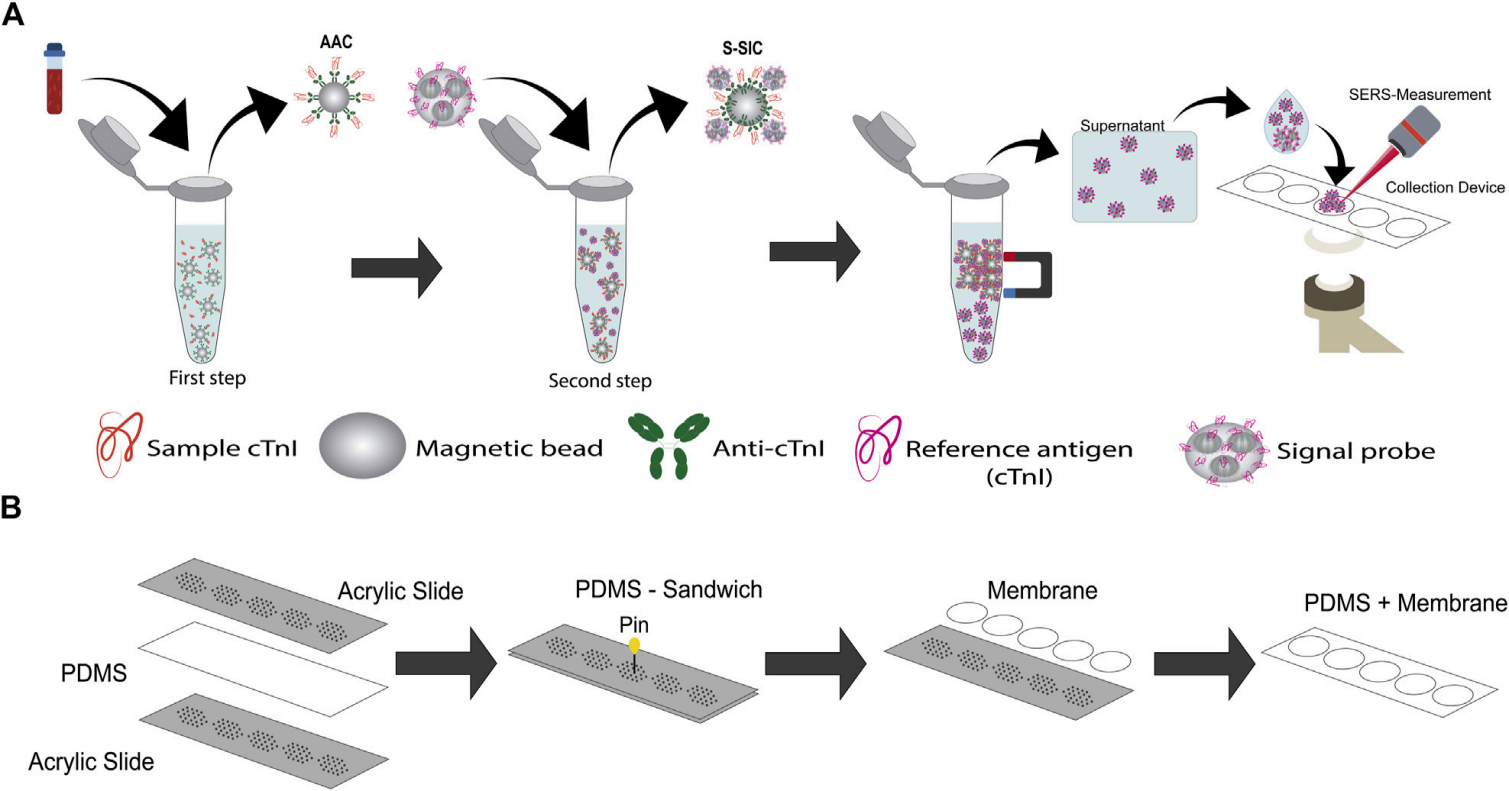
SERS-based competitive immunoassay proposal by **(A)** Garza and Cote (2017) under the principle of sequential saturation, adapted from Garza and Cote (2017), homemade collection device used for the SERS experiments in incise **(B)**.

Lin et al. (2021a) explored the quantification of cTnI under the competitive immunoassay format. The signal probes consisting in Au-Ag core-shell nanodumbbells modified with the reporter molecule and the reference antigen (cTnI-4MBA-Au@Ag) are mixed and incubated with the capture probes, which were formed by magnetic nanoparticles functionalized with a polydopamine (PDA) layer, Au nanoparticles, and a thiolate aptamer of cTnI (apt-AuNPs@PDA@Fe_3_O_4_), in the presence and absence of target cTnI. Once the antigen-aptamer complexes were formed, the magnetic probes were collected with the help of a magneto, then washed, and dispersed in PBS. A certain volume of this suspension was placed on the surface of the glass slide and dried. Then the Raman spectra were collected. A decrease in the SERS signal of the selected 4MBA peak reflects the presence of target cTnI, due to decreased magnetic nanoparticle surface area of signal probes containing the labelled cTnI. The decrease in the SERS signal showed a linear relationship with the antigen concentration. In this work, the precision of the aptasensor was evaluated through its repeatability, from the relative standard deviation (RSD) of 15 Raman intensity measurements of a certain point (RSD of 3.50%). The stability at 1, 5, 15, 30, and 60 days was corroborated by the RSD value of 3.50% obtained. Using a competitive immunoassay format under the principle of sequential saturation, Garza and Cote (2017) detected cTnI by SERS (Figure 7A). The competitive immunoassay included the mixture and incubation during 20 min of the sample containing cTnI with a solution of capture probes, anti-cTnI functionalized magnetic beads (anti-cTnI@Fe_3_O_4_) to form the AAC. The above process allowed reaching the equilibrium state before the addition of the signal probes (cTnI-SiO_2_@DTNB@Ag), to fill the remaining binding sites on the magnetic beads. The signal probes (aggregates of silver nanoparticles functionalized with DTNB encapsulated with silica and functionalized with the antigen reference cTnI), were added in a known amount, to the unlabelled cTnI-capture probes complexes, and incubated for 10 min. Once the immunoassay was completed, the magnetic beads were separated with a magnetic field, and the supernatant was collected for SERS measurements that were carried out in a homemade collection device (Figure 7B). The collection device was constructed as follows: between two rectangular acrylic slides with the same pattern of clustered holes, a thin layer of polydimethylsiloxane (PDMS) with the same dimension of the slides was placed. Once the holes of both slides exactly matched, each hole was crossed by a needle. After that, the top slide was carefully removed, and membranes of 20 nm pore size were placed on the top of the PDMS surface to cover the holes. A volume of the supernatant was placed on the membrane and vacuum was applied below the slide to suck out the liquid; in this way the magnetic beads were concentrated in a specific area where the SERS measurements are conducted. SERS spectra were measured at different points covering the collection spot and the Raman intensity of the band mark in all the spectra was averaged to obtain a single value at each cTnI concentration. In this assay, the SERS signal of the free label ligands that did not participate in the competitive immunoassay increases in a non-linear way as cTnI concentration increases from 0 to 250 ng/ml. This behaviour is because the free label ligands in the supernatant are gathered on the collection device and their measured SERS intensity is proportional to the amount of nanoprobes present, which in turn is proportional to the amount of analyte in the sample.

**FIGURE 8:**
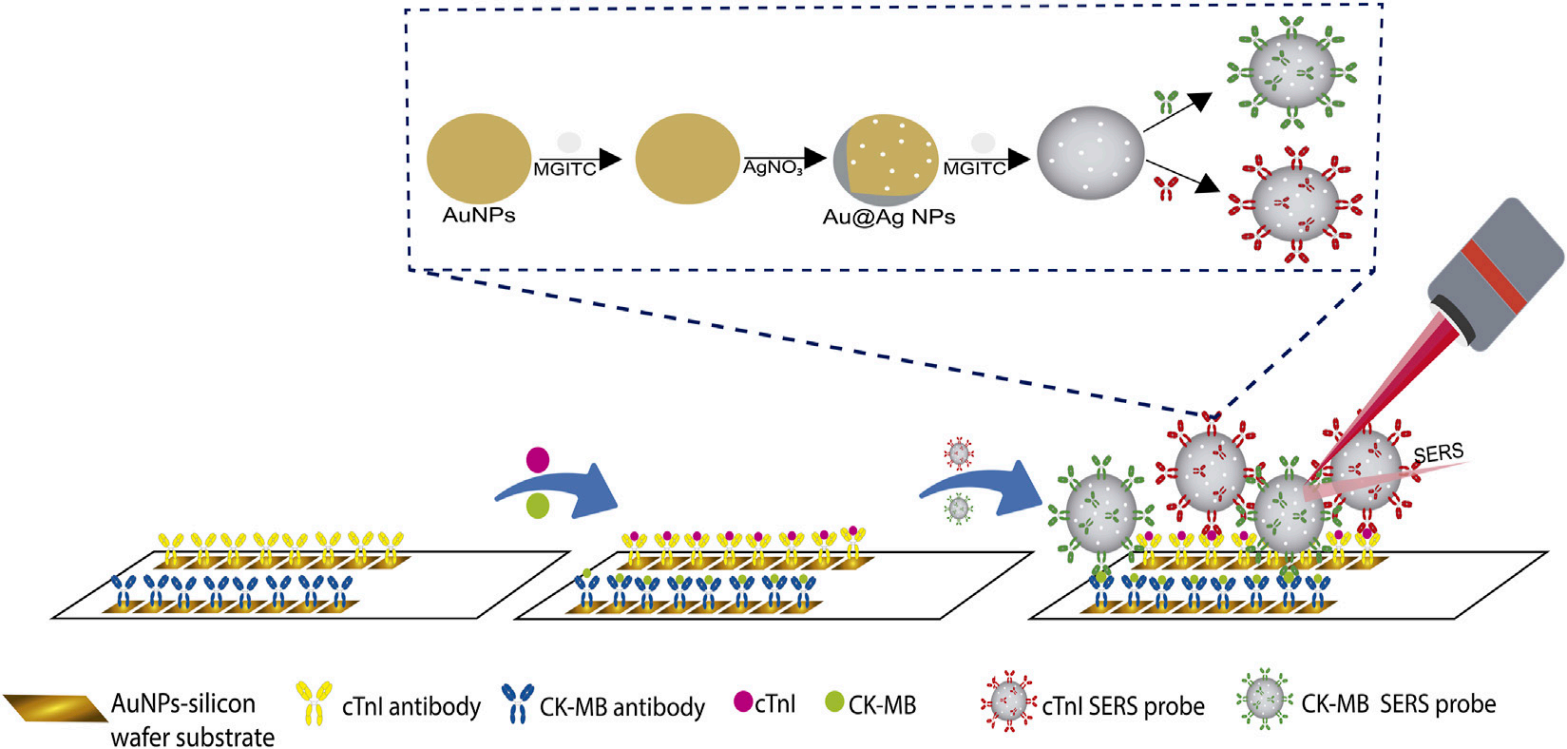
Schematic representation of SERS-based sandwich immunoassay for the independent quantification of cTnI and CK-MB, adapted from Cheng et al. (2019).

Wang J. et al. (2021) developed a microcavity SERS substrate on which the simultaneous detection of cTnI and CK-MB was conducted using a sandwich immunoassay. Polystyrene (PS) microspheres that contained microcavities produced during the modification with gold nanoparticles, were fixed on silicon wafer substrates. To form the capture substrate, monoclonal antibodies of cTnI and CK-MB were immobilized onto the surface of this template. The detection probes were constructed using AuNPs modified with the corresponding antibodies and Raman reporter molecules specific for each antigen. The SERS spectra containing information of the RRMs were obtained by measuring ten random spots of the immune substrate. The recovery rates of cTnI and CK-MB ranged from 94.9% to 121.6% and the inter assay variation did not exceed the coefficient of variance (CV) of 15%. Su et al. (2018) developed a SERS sensing platform with a macroporous trimetallic array on an ITO slice (Au@Ag@Au@ITO substrate), and used it for the simultaneous multiplexed detection of three biomarkers involved in the cardiorenal syndrome, cTnI, NT-ProBNP, and neutrophil gelatinase-associated lipocalin (NGAL). The Au@Ag@Au@ITO substrate elaboration comprised the successive electrochemical deposition of Au and Ag on PS sphere template-modified ITO slices. After that, the PS template removal was induced followed by the ion-beam sputtering deposition of Au. The multiplex detection was made under a sandwich immunoassay format; therefore, the developed substrate was modified with the monoclonal antibody of each evaluated antigen to obtain the capture substrate. Three SERS detection probes were used based on Ag−Au nanostars, each one modified with the polyclonal antibody and the Raman reporter molecule selected for each antigen. The simultaneous antigen detection was achieved tracking one peak of each reporter molecule in the obtained spectrum. The proposed immunosensor proved to be stable after 4 weeks, it does not present significant cross-reactivity among the tested antigens, and the inter-assay reproducibility was good with a CV of 11.2% for cTnI. Cheng et al. (2019) quantified cTnI and CK-MB independently using the SERS-based sandwich immunoassay depicted in Figure 8. AuNPs were deposited on silicon wafer substrates, which were then arranged to form two rows on a glass slide. Each row was covered with rabbit anti-cTnI monoclonal antibody or with rabbit anti-CK-MB monoclonal antibody to obtain the capture substrates. The sandwich ELISA immunoassay was completed in each capture substrate of the template using a unique detection probe for both biomarkers that consisted in Au@Ag core–shell nanoparticles with MGITC as the Raman reporter molecule. The Raman intensity increased in a non-lineal way with the increase in biomarker concentration in the range of 0–100 ng/mL. A good cTnI recovery percentage of 97.3% and a CV value of less than 16% between biological replicates were reported.

**FIGURE 9:**
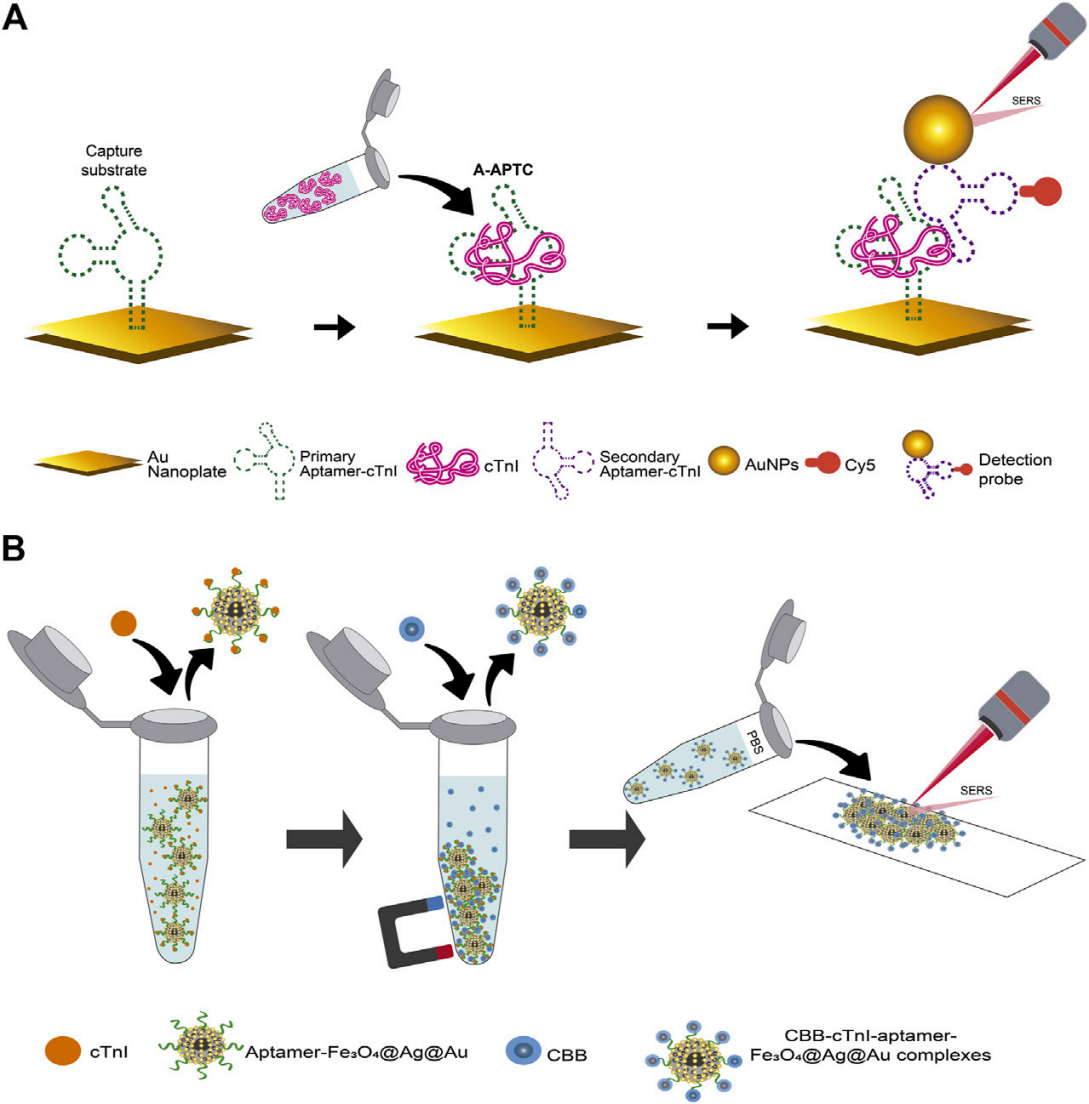
Schematic representation of aptamer-based SERS assays for cTnI: **(A)** the sandwich assay using an atomically flat Au nanoplate as the plasmonic substrate, adapted from Lee et al. (2020), **(B)** assay using the Bradford method, adapted from Lin et al. (2021b).

Lee et al. (2020) reported the detection of cTnI using an aptamer-based SERS sandwich assay. The capture substrate was an atomically flat Au nanoplate functionalized with the primary aptamer of cTnI. Likewise, AuNPs functionalized with the secondary cTnI aptamer that contained the Cy5 reporter molecule constituted the detection probes. The detection procedure is schematized in Figure 9A and comprises the evaluation of a specific concentration of cTnI added to a hybridization buffer solution with or without 20% human serum. The solution was added on the capture substrate to provoke the antigen-aptamer complex formation (A-APTC). After a washing process, the detection probes were added to form the sandwich-shape immunocomplexes. After a washing and drying process, the SERS experiments on the Au nanoplates were conducted. Regarding to the specificity of the assay it was disclosed that proteins in serum such as IgG and albumin may induce background SERS signals. In the work of Lin et al. (2021b) the quantification of cTnI was carried out by taking advantage of the strong Raman signal and the ability of the Coomassie brilliant blue dye (CBB) to binding basic amino acids of proteins in acidic media, as well as the high selectivity of aptamers to bind particular antigens. This novel quantification strategy is depicted in Figure 9B and comprises the addition of the sample (human serum without dilution and no pretreatment) into an Eppendorf tube containing Fe_3_O_4_@Ag@Au nanoparticles modified with the sulfhydryl28-mer aptamer. After ultrasonication, incubation, and washing, the Raman reporter molecules (see Table 3) were added and incubated during 10 min to bind the cTnI that was retained by the capture probes through its interaction with the amino acids present in the antigen. Once the Raman reporter molecule-cTnI-aptamer-Fe_3_O_4_@Ag@Au complexes were formed, these were recovered using magnetic separation, washed, and re-suspended in PBS. Finally, for the Raman experiments the re-suspended solution was measured on the surface of the glass slide placed on the magnet in parallel using the dry form. Regarding stability, the SERS results of the analyte after reaction with the reporter molecule are consistent only within 5 days after preparation. To prove the accuracy of the detection method, a recovery test based on the standard addition method was conducted in healthy human serum sample by spiking different concentrations of the cTnI target. The recovery rate was found to be between 92 and 115%, and RSD (n = 3) from 7.4 to 12.7%. Other analytical parameters of interest for POCT extracted from the discussed articles are found in Table 3.

#### 6.2.1 Microfluidic devices

A microfluidic system is based on micron-sized channels where fluid is transported continuously on a substrate (Luka et al., 2015). The use of microfluidic platforms in biosensing provides unique advantages, such as the possibility of automation, enhancement of mass transport and integration of the whole mechanical processes involved in the immunoassay (such as washing and mixing) in a single chip (lab-on-a-chip, LoC). Microfluidic systems can be active or passive, depending on how the flow is produced. In active systems, the fluid transport is achieved using actuators or external power supplies, like when assisted by pumps. Passive systems operate without a power source, rather taking advantage of natural phenomena like capillary pressure, surface tension or gravity, resulting in simpler devices (Kant and Abalde-Cela, 2018; Narayanamurthy et al., 2020; Shi et al., 2021). Therefore, when fast and highly sensitive techniques such as SERS are integrated with a microfluidic system, there is a real possibility to obtain true POCT devices.

**FIGURE 10:**
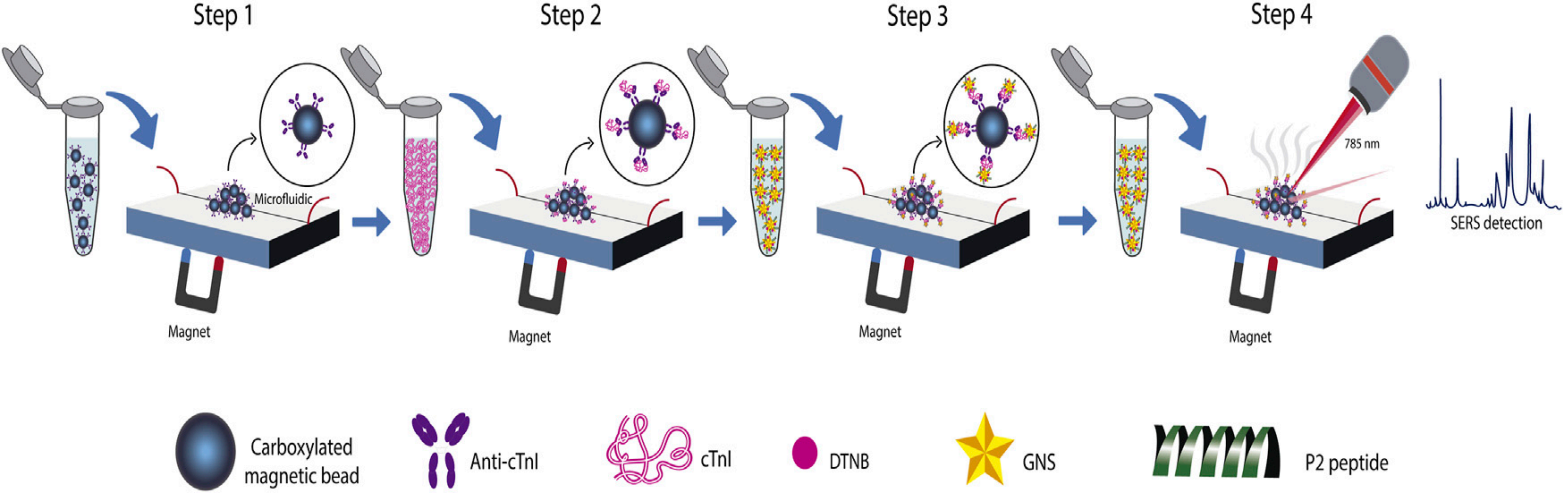
Schematic representation of the sandwich immunoassay integrated in an active microfluidic device for the quantification of cTnI and neuropeptide Y, adapted from Wen et al. (2020).

For the SERS detection of cTnI together with neuropeptide Y, the latter also being a biomarker involved in myocardial infarction, Wen et al. (2020) developed a sandwich immunoassay integrated with an active microfluidic device. Magnetic beads conjugated with the corresponding polyclonal antibody were the capture probes. In this work, two SERS tags were designed by varying the Raman reporter molecule that was conjugated on the surface of gold nanostars. Later, the SERS tags were modified with a short peptide biorecognition element (BRE) specific to each antigen to create the detection probe. The BREs were identified using *in silico* methods and evolved from a starting parental affinity peptide identified from a phage display peptide library (Xiao et al., 2018). In the case of detection of cTnI, the BRE was identified as the P2 peptide, a small size peptide (∼3 nm) able to bind residues 114-141 of troponin with high affinity and without competing with the target site of the antibody on the capture probes. Figure 10 shows the schematic representation of the setup and process for the multiplex immunoassay analysis proposed. The quantification process includes four steps: 1) formation of a uniform monolayer of capture probes on the sample chamber with the aid of a magnet; 2) addition and incubation of the sample with the formed magnetic beads film; 3) addition and incubation of the SERS detection probes to the antigen-antibody magnetic beads complexes. In this step, the microfluidic devices are positioned on a stir plate to facilitate the mixing between sample and magnetic beads; 4) drying of the microfluidic device for 10 min at room temperature and making of Raman experiments. The advantage of using a microfluidic system was reflected mainly in the generation of a reusable analytical device (after a repeated washing process). On the other hand, the microfluidic platform was simple and comprised only one inlet and one outlet, as well as a sample chamber for incubation of samples, mixing, and evaluation of biomarkers. The precision of the technique was evaluated by the inter-assay coefficient of variation analyzing patient samples, where a CV of less than 10% was obtained, corresponding to a precise methodology.

#### 6.2.2 Paper-based devices

Lateral flow immunoassays (LFIA) and microfluidic paper-based analytical devices (μPAD) are the variants of paper-based devices, the most tackled by scientists for the development of POC testing (Lim et al., 2019). Nowadays, LFIA are popular in the medical diagnosis market as rapid tests for pregnancy and for the diagnosis (and in some cases the prognosis) of various diseases including cancer, HIV and COVID-19 (Andryukov, 2020; Sachdeva, Davis and Saha, 2021). LFIA test strip comprises four generic elements: 1) sample pad (SP); here occurs the first LFIA-sample interaction and in some cases the sample pretreatment. The adequate flow by capillary action and the integrity of the sample through the device is ensured in this zone. 2) conjugate pad (CP); depending on the immunoassay format used in the LFIA, particles (mainly gold nanoparticles or latex microspheres) are placed on the CP. These particles are conjugated with: 1) the antigen (known as reference antigen) in the case of indirect competitive format, or 2) the antibody (called antibody of detection or secondary antibody for the sandwich format, or reference antibody for the direct competitive format). 3) reaction membrane, composed by the test line (T-line) and control line (C-line). In the sandwich format, the capture or primary antibody is immobilized on the T-line to allow the formation of a sandwich-type immune complex. Recognition of the analyte (antigen) is carried out through a change in color or the fluorescence response of the T-line. In the competitive assay, a reference antigen or a reference antibody is immobilized on the T-line for the direct or indirect detection, respectively. Thus, the unlabeled analyte coming from the sample (antibody or antigen) is detected by its capacity to compete with the conjugated nanoparticle in the CP. Finally, a reference antigen is immobilized on the T-line in the indirect format. The binding of the reference antigen with the antibody of the sample is tracked through the detection of the secondary antibody of the conjugate in the CP. At the C-line, complementary antibodies, mainly IgG (mouse, goat, or rabbit) are generally immobilized, having the function of indicating that the lateral flows are effective, and that the antibodies are active. 4) absorption pad (AP); this element collects the excess liquid sample and avoids a back flow (Koczula and Gallotta, 2016; Di Nardo et al., 2021; Liu et al., 2021; Majdinasab et al., 2022). A μPAD is the result of including a passive microfluidic system in a paper-based assay; here, some form of patterning is produced by hydrophobic materials on the porous base of the paper substrate to create flow channels where the sample goes through (directed flow). Therefore, automation and integration can add to the benefits of paper-based devices, such as low cost, rapid detection, ease of handling, and even operation by unskilled personnel. Despite of the great improvements introduced in the area of LFIA and μPAD (Andryukov, 2020; Boobphahom et al., 2020), the information provided by this kind of devices is usually dichotomous (they just indicate the presence or absence of the analyte). The obtainment of quantitative information can substantially enhance the efficiency of these devices, and as a consequence, increase their practical use (Urusov et al., 2019). The above can be achieved by coupling the paper-based devices with techniques such as SERS; the same gold nanoparticle conjugates used in the CP can also be employed as the plasmonic source for the acquisition of the SERS signal at the T-line. Furthermore, multiplex detection using SERS is possible even using a single T-line.

**FIGURE 11:**
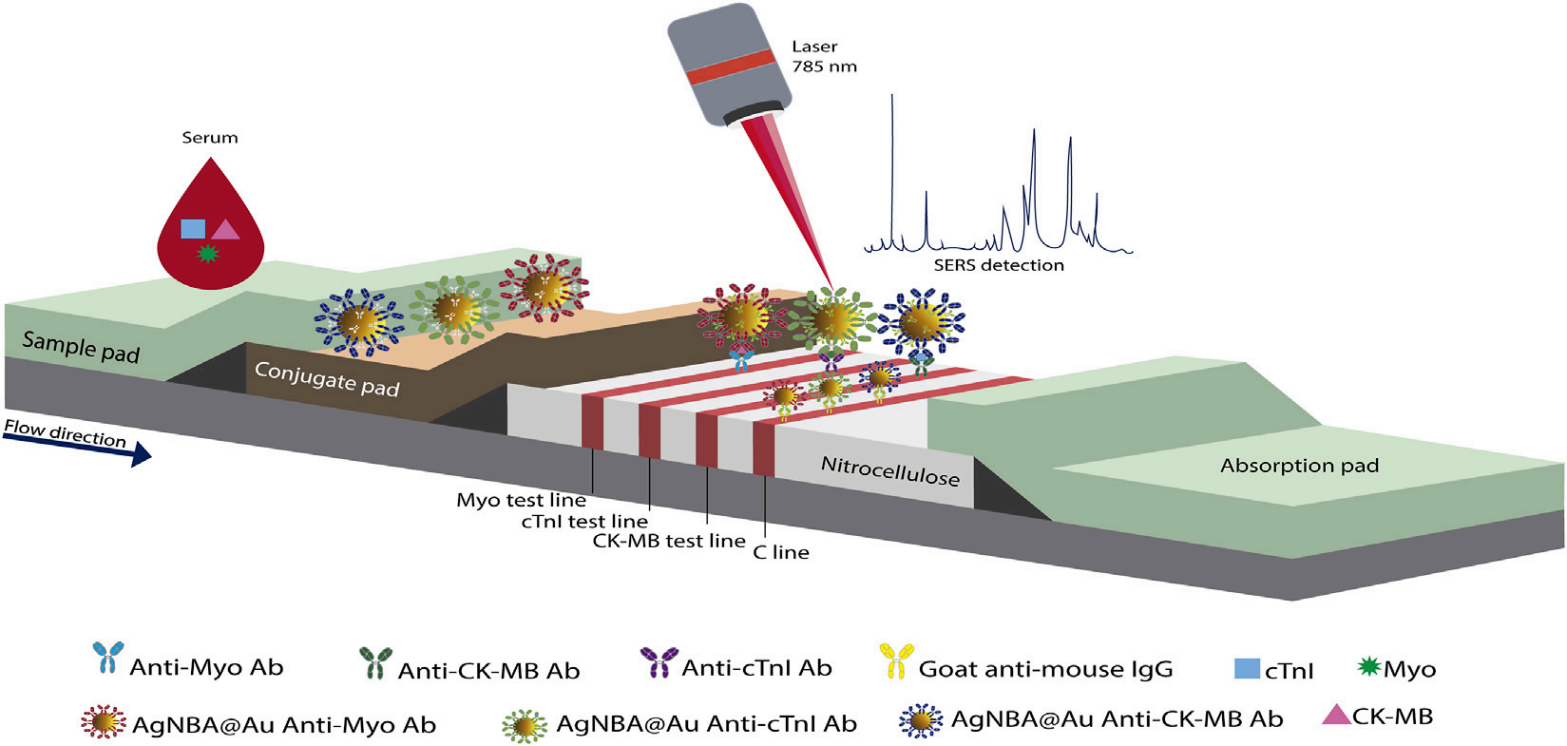
Schematic representation of the multiplex SERS-based lateral flow assay proposed by Zhang et al. (2018a).

Among works on the detection of cTnI by SERS-based on LFIA is the proposal by Zhang et al. (2018a). They detected cTnI, myoglobin (Myo), and kinase-MB isoenzyme (CK-MB) in different T-lines as shown in Figure 11. Three detection probes were mixed in the CP. These probes were constituted by silver-gold core-shell nanoparticles with the NBA encapsulated between the two metals (AgNBA@Au), but the conjugated mAb changed depending on the antigen to be quantified. Three test lines and one control line conformed the LFIA and were sprayed with monoclonal detection antibodies against each antigen and goat anti-mouse IgG, respectively. Raman measurements were carried out at each test line after the flow of the sample from the sample pad to the absorption pad, and after the control line presented a color change. In order to increase the homogeneity of the SERS experiments (due to the fluctuation of Raman intensities by the intrinsic lack of homogeneity of the nitrocellulose membrane that conforms the LFIA), maps were generated from Raman spectra acquired on each test line over a 210 μm × 210 μm area, with a total image acquisition time of about 7 min. The Raman intensities of 441-pixel points (1pixel = 10 μm × 10 μm) were averaged to obtain a reproducible intensity value. The inter-batch variability was less than 8.5%, which means an acceptable precision of the SERS-based LFIA. The cross-reactivity among the three biomarkers was negligible.

**FIGURE 12:**
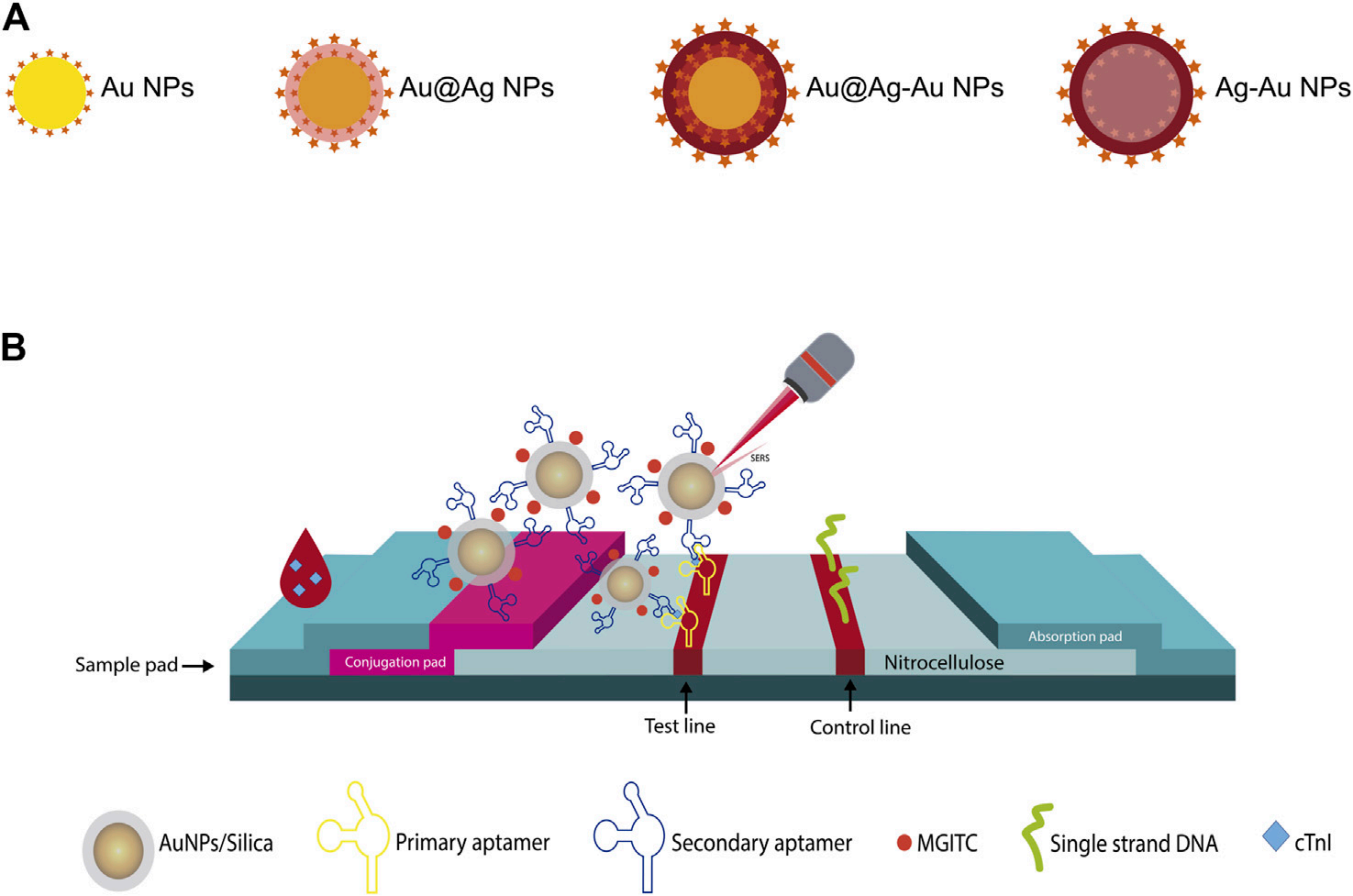
**(A)** The four different types of SERS substrate used to elaborate the capture probes in the SERS-based LFIA strip presented by Bai et al. (2018), **(B)** Schematic representation of the aptamer-based SERS assay on paper platform, adapted from Tu et al. (2020).

In a later work published by these authors, the same antigens were detected with a similar LFIA strip, but now simultaneously using a single test line (Zhang et al., 2018b). This was achieved by using the same detection probes, but the Raman reporter molecules embedded in the Ag@Au nanoparticles were specific to each antigen. In the single test line, the three capture antibodies specific to each target antigens (cTnI, CK-MB and Myo) were jetted. From maps generated by Raman spectra obtained within an area of 200 μm × 200 μm, with 10 min of signal acquisition, the distinction and quantification of the analytes was achieved. The above was possible when monitoring the Raman intensity of a characteristic peak of the reporter molecule used for each biomarker. This single T-line proposal allowed the optimization of the required time to obtain quantitative information of the three tested antigens (see Table 3). However, the Raman signal acquisition time is long compared with a conventional Raman measurement. No overlap on the chosen peaks of the reporter molecules was observed, which allowed the use of raw SERS spectra for quantitative analysis, without the need of a deconvolution treatment. The CV was 9.2% among different batches, and the cross-reaction of the three biomarkers was also negligible. Another approach for the quantitative analysis of cTnI using SERS-based LFIA strip was presented by Bai et al. (2018). To improve the LFIA sensitivity, the authors evaluated four capture probes that were formed by conjugating the RRM and the anti-cTnI antibody on the SERS substrates, as represented in Figure 12A. These probes were constituted by citrate-capped NPs (Au NPs, Au core with Ag shell NPs (Au@Ag NPs), rattle-like Au core in Ag-Au shell NPs (Au@Ag-Au NPs), and Ag-Au NPs). The capture probe that yielded better results was that based on Au@Ag-Au NPs. The test and control zone of the proposed LFIA strip contained the capture antibody and goat anti-mouse IgG, respectively. The Raman spectra on the test line was averaged from five different positions. In the presence of cTnI, the peak at 592 cm^−1^ increases gradually with increasing the antigen concentration. The SERS-based lateral flow immunoassay for cTnI developed by Khlebtsov et al. (2020) considered the use of a novel SERS substrate for the elaboration of the detection probes. The detection probe used in this study consisted of anti-cTnI monoclonal antibodies and clone IC4-conjugated gold nanorods (AuNRs)-gold core-shell with the reporter molecule NBT encapsulated between the two metals (mAb-AuNRs@NTB@Au core-shell nanoparticles). In the LFIA device, the mAb-AuNRs@NTB@Au core-shell nanoparticles were placed on the conjugated pad. In order to create the test and control zones, anti-сTnI mAb, clone IC19 and goat anti-mouse immunoglobulin (GAMI) antibodies were used. cTnI was identified and quantified by SERS mapping of the test zone over a 400 μm × 400 μm area. However, the Raman intensity values used to construct the calibration curve were obtained from the averaged SERS spectra of the test zone maps, integrated over the spectral band from 1,280 to 1,360 cm^−1^.

On the other hand, the proposal of Tu et al. (2020) included an aptamer-based lateral flow immunoassay for monoplex detection of cTnI. Figure 12B displays the schematic diagram of the aptamer-based paper strip. To improve the performance of the LFIA, a chase buffer was used to facilitate the capillary migration of the sample along the strip, without dilution requirements. The sample was introduced onto the sample pad followed by the chase buffer. The detection probes that consisted of MGITC functionalized AuNPs encapsulated in silica shell and conjugated with a secondary aptamer (aptamer/silica/MGITC/AuNPs) were stored in the conjugate pad. A biotinylated primary aptamer of cTnI and a biotinylated single-strand DNA (the reverse complimentary DNA strand of the secondary cTnI aptamer) were immobilized in the test and control region, respectively, through biotin–streptavidin interaction. Therefore, when the antigen-detection probes reached the test region, the formation of sandwich-shaped immunocomplexes occurred. The excess of detection probes continued to flow and those without cTnI were bound to the single-strand DNA in the control line. The SERS signal obtained on the test line was the average of the intensity of three measurements taken by moving from the left to the right side. This LFIA proposal presented a recovery rate higher than 93.8% in human serum samples and a good stability when stored at room temperature over 10 days, attributes than can be influenced by the use of aptamers (Zhu et al., 2015).

## 7 Conclusion and outlook

POCT for the detection of cardiac biomarkers such as cTnI are among the most developed and commercially available. For cTnI, there is a high demand for those tests that provide quantitative information, can be used in hospitals and primary health care, and exhibit the possibility of being adapted to centralized clinical laboratories. Herein, we explore the use of SERS to detected cTnI and from this information, some of the main aspects to consider in the development of a SERS-based POCT for cTnI can be brought to light: 1) Due the low Raman cross-section of cTnI, the label based approach seems to be the best option for achieving multiplex and quantitative information in a wide range of sample concentrations 2) There is not a specific route to follow regarding the best selection of the analytical strategy to obtain the ideal POCT to cTnI. Due to its high specificity and practicality, the design of a sandwich-type SERS immunoassay is commonly used; however, the Raman measurements can be carried out in solution or statically on the surface of substrates. If the solution mode is chosen, despite of the disadvantage of low experimental reproducibility by continuous movement of the plasmonic material, the Raman measurements can be performed in the same container where the immunoassay reaction was completed, compensating the experimental error. An improvement in this area is the use of magnetic beads which can be fixed on some point of the container to directly record the Raman experiments or integrate the system to a microfluidic chamber. If the statically mode is selected, a better control of the experimental data can be achieved, but washing, mixing or evaporation steps inherent to the analytical process make more complex the proposal. Improvements are achieved integrating microfluidics or paper-based platforms. Integration of microfluidic chip platforms can meet the requirement of POCT automation specially for multiplex analysis. However, total automation in each step of the immunoassay still depends on the use of active pumps and valves, resulting in a complex analytical system. On the other hand, SERS LFIA strips are recognized to be low cost and give quick results. The use of a large volume of sample and the high variability in the obtained Raman signal values in both intra and inter-assay studies are still big challenges due to the fluctuation of Raman intensities because of the intrinsic lack of homogeneity of the nitrocellulose membrane that conforms the LFIA. Combining SERS-LFIA with passive microfluidics and inclusion of confocal SERS mapping could be a promising approach to avoid these problems at experimental level. 3) The use of antibodies as recognition elements by taking advantage of the antigen-antibody interaction turns out to be the best option as long as the following is considered: the use of more than one antibody to ensure the recognition of free and complexed cTnI is preferable. Despite the availability of a wide variety of antibodies to cTnI (pAbs, mAbs, and rAbs), a recipe of how to achieve the best combination is not yet available; however, this route should compensate the inherent immunoassay problems that result in variability of the measured cTnI concentration. These problems are enlisted as follows: 1) presence of endogenous or exogenous molecules that causes structural modifications of the antibody or of the cTnI (post-translational, proteolytic degradation, phosphorylation, oxidation); 2) cross-reactivity of antibodies with troponin isoforms; 3) Proteolytic degradation in cTnI, especially in the external N- and C-terminal regions, that impacts in its binding with antibodies through epitopes located at the external regions; 4) presence of autoantibodies for cTnI that decrease the accessibility of the biomarker to join its antibody; 5) presence of heterophile antibodies in the blood sample that could cause false-positive or -negative results. A proposal to tackle the deficiencies of antibodies from a genetic perspective is using aptamers; however, they are at an early stage of research, so more results on their use will confirm their advantages and drawbacks. 4) In the most discussed literature, the reported relative standard deviation was high (low accuracy). This could be a serious problem mainly for the diagnostic and control of diseases like AMI, where the differences in concentration as a function of time show subtle changes, or in diseases where the cut-off is established as a specific value or a narrow range. 5) so far, despite the fact that detection of other analytes by SERS in body fluids such as saliva and sweat has been proven, the quantitative detection of cTnI by this technique has not been reported, even when it is known that this biomarker is also present in these matrices (Senf et al., 2020; Arabi et al., 2021; Hao et al., 2021; Zheng et al., 2021; Constantinou, et al., 2022; Singh et al., 2022). This application is of great importance because it would open a window of possibilities for the non-invasive detection of cTnI by SERS.
